# A New Species of *Garjainia* Ochev, 1958 (Diapsida: Archosauriformes: Erythrosuchidae) from the Early Triassic of South Africa

**DOI:** 10.1371/journal.pone.0111154

**Published:** 2014-11-11

**Authors:** David J. Gower, P. John Hancox, Jennifer Botha-Brink, Andrey G. Sennikov, Richard J. Butler

**Affiliations:** 1 Department of Life Sciences, The Natural History Museum, London, United Kingdom; 2 Evolutionary Studies Institute, University of the Witwatersrand, Johannesburg, South Africa; 3 Karoo Palaeontology, National Museum, Bloemfontein, South Africa and Department of Zoology and Entomology, University of the Free State, Bloemfontein, South Africa; 4 Borissiak Paleontological Institute, Russian Academy of Sciences, Moscow, Russia; 5 Kazan Federal University, Kazan, Russia; 6 School of Geography, Earth and Environmental Sciences, University of Birmingham, Birmingham, United Kingdom; 7 GeoBio-Center, Ludwig-Maximilians-Universität München, Munich, Germany; Raymond M. Alf Museum of Paleontology, United States of America

## Abstract

A new species of the erythrosuchid archosauriform reptile *Garjainia* Ochev, 1958 is described on the basis of disarticulated but abundant and well-preserved cranial and postcranial material from the late Early Triassic (late Olenekian) Subzone A of the *Cynognathus* Assemblage Zone of the Burgersdorp Formation (Beaufort Group) of the Karoo Basin of South Africa. The new species, *G. madiba*, differs from its unique congener, *G. prima* from the late Olenekian of European Russia, most notably in having large bony bosses on the lateral surfaces of the jugals and postorbitals. The new species also has more teeth and a proportionately longer postacetabular process of the ilium than *G. prima*. Analysis of *G. madiba* bone histology reveals thick compact cortices comprised of highly vascularized, rapidly forming fibro-lamellar bone tissue, similar to *Erythrosuchus africanus* from Subzone B of the *Cynognathus* Assemblage Zone. The most notable differences between the two taxa are the predominance of a radiating vascular network and presence of annuli in the limb bones of *G. madiba*. These features indicate rapid growth rates, consistent with data for many other Triassic archosauriforms, but also a high degree of developmental plasticity as growth remained flexible. The diagnoses of *Garjainia* and of Erythrosuchidae are addressed and revised. *Garjainia madiba* is the geologically oldest erythrosuchid known from the Southern Hemisphere, and demonstrates that erythrosuchids achieved a cosmopolitan biogeographical distribution by the end of the Early Triassic, within five million years of the end-Permian mass extinction event. It provides new insights into the diversity of the Subzone A vertebrate assemblage, which partially fills a major gap between classic ‘faunal’ assemblages from the older *Lystrosaurus* Assemblage Zone (earliest Triassic) and the younger Subzone B of the *Cynognathus* Assemblage Zone (early Middle Triassic).

## Introduction

Following the end-Permian mass extinction event, archosauromorphs (the clade comprising all reptiles more closely related to birds and crocodilians than to lepidosaurs: [1]) underwent a major evolutionary radiation in the Early Triassic, and ecologically replaced therapsids in many niches, including those of largest terrestrial predators in continental ecosystems (e.g. [2]–[8]). The fossil remains of these large predators are typically rare, isolated and fragmentary (e.g. [9]). The generally incomplete nature of their fossils has created challenges for early archosauromorph systematics, and this is partly reflected by the fact that the vast majority of named genera are monotypic and known from relatively small spatiotemporal distributions (e.g. [10]). Compounding this, non-crown-group archosauromorphs have in general been less studied than their crown-group relatives, even for taxa represented by much better fossil material such as the South African *Proterosuchus* Broom, 1903 [11].

Erythrosuchidae Watson, 1917 [12] is a family of generally large, carnivorous, non-archosaurian archosauriform (*sensu* [13]) diapsid reptiles known from the Olenekian (late Early Triassic) to Anisian (early Middle Triassic) or Ladinian (late Middle Triassic) of Pangaea (e.g., [9]–[10], [14]). Erythrosuchid anatomy and systematics is generally weakly characterised, with uncertainty remaining over the taxonomic content, phylogenetic relationships, and palaeobiology of the family ([10]). The best-known erythrosuchids are *Erythrosuchus africanus* Broom, 1905 [15] from the early Middle Triassic (Anisian) Burgersdorp Formation (Subzone B of the *Cynognathus* Assemblage Zone) of South Africa, *Garjainia prima* Ochev, 1958 [16] from the late Olenekian Yarenskian Gorizont of European Russia, and *Shansisuchus shansisuchus* Young, 1964 [17] from the Anisian Ermaying Formation of China. However, the morphology of only the former of these has been documented in any detail for most parts of its skeleton ([18]–[20]).

**Figure 1:**
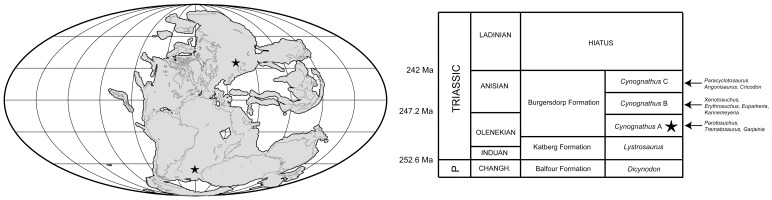
Early Triassic palaeomap and South African Lower–Middle Triassic stratigraphy. Palaeomap shows the localities of *Garjainia prima* (in modern day Russia) and *Garjainia madiba* sp. nov. (in modern day South Africa) on a 240 Ma continental reconstruction (generated using tools at www.fossilworks.org created by John Alroy). Simplified stratigraphic column shows the level yielding *Garjainia madiba* sp. nov. (marked by a star), and key taxa known from the *Cynognathus* Assemblage Zone.

Here we describe a new species of erythrosuchid from the late Early Triassic (Olenekian) of South Africa, and document its anatomy and bone histology. This new species comes from Subzone A of the *Cynognathus* Assemblage Zone, within the lowermost part of the Burgersdorp Formation (Fig. 1), and is therefore older than *E. africanus* from the middle levels (Subzone B [21]) of the same Assemblage Zone in the main Karoo Basin. The new erythrosuchid has been mentioned previously by Hancox et al. [21] and Hancox [22], among others, and was tentatively referred to the genus *Garjainia* by Hancox [22]. Here we formally refer the new species to *Garjainia*.

## Material and Methods

All fossil specimens described herein or used for comparative purposes are curated in recognised repositories and were accessed with the explicit permission of the appropriate curators and/or collection managers. No permits were required for the described study, which complied with all relevant regulations. See Text S1 for list of comparative material and sources of information. Repository locations and abbreviations for all specimens discussed in the text are as follows: BMNH, The Natural History Museum, London; BP, Evolutionary Studies Institute (formerly Bernard Price Institute for Palaeontological Research), University of the Witwatersrand, Johannesburg, South Africa; NM, National Museum, Bloemfontein, South Africa; PIN, Palaeontological Institute, Moscow, Russia.

### Nomenclatural Acts

The electronic edition of this article conforms to the requirements of the amended International Code of Zoological Nomenclature, and hence the new names contained herein are available under that Code from the electronic edition of this article. This published work and the nomenclatural acts it contains have been registered in ZooBank, the online registration system for the ICZN. The ZooBank LSIDs (Life Science Identifiers) can be resolved and the associated information viewed through any standard web browser by appending the LSID to the prefix ‘‘http://zoobank.org/’’. The LSID for this publication is: urn: lsid:zoobank.org: pub: 6209DE64-AFB2-46E5-A04D-859628BBF47F. The electronic edition of this work was published in a journal with an ISSN, and has been archived and is available from the following digital repositories: PubMed Central, LOCKSS.

### Terminology and Nomenclature

*Vjushkovia triplicostata* Huene, 1960 [23] is here accepted as a subjective junior synonym of *Garjainia prima* Ochev, 1958 [16], following Gower & Sennikov [9]. *Shansisuchus heiyuekouensis* Young, 1964 [17] is considered a subjective junior synonym of *S. shansisuchus* Young, 1964 [17], following Charig & Reig ([24]: 164–165), Charig & Sues ([14]: 36), Gower ([18]: 360) and personal observation (DJG).

The orientation of limb bones follows that of Gower ([20]: 55). We follow the terminology for vertebral laminae outlined by Wilson [25], based upon the basis of the homologous structures that they connect, and the associated terminology for vertebral fossae proposed by Wilson et al. [26].

We use the English-based transliteration “Ochev” rather than the German “Otschev” for the Russian name Ochev. Both versions have been published elsewhere (e.g., compare [9] and [27]). Colours reported for sediments are based on direct comparisons with a Munsell system chart.

### Bone Histology

Bone microstructure records information about the life history of vertebrates, such that insights into lifestyle, biomechanics and growth can be obtained for extinct animals that would otherwise not be possible using more traditional methods. We analysed the bone microstructure of seven limb bones from individuals of various sizes (and likely ontogenetic ‘stages’). These elements (Table 1; Text S1) were referred to the new species on the basis of overall morphological similarity, and our identifications were supported by these elements coming from localities that are not known to yield other archosauromorph reptiles of a similar size. Because the new species is incompletely known and histological analysis is a destructive sampling technique, only fragmentary elements were thin sectioned. However, the examination of several elements from several individuals has allowed us to document some inter-elemental as well as inter-individual variation in this taxon. Precise assessments of ontogenetic age for each sectioned element are not possible.

**Table 1:** Quantifiable characters for the *Garjainia madiba* sp. nov. elements thin sectioned in this study.

Specimen	Element	MW	TBA	Vasc	AvPOD	Growth	K	C
BP/1/		mm	mm^2^	%	µm	Marks		
7129	humerus	28	401.8	18.1	159	2	0.5	0.852
7219	humerus	22.2	475.9	17.6	163	2	0.2	0.824
7217	femur	36.8	643.5	10.9	182	2	0.42	0.812
6232j	femur	54.1	1817.2	27.3	204	3	0.57	0.489
7216	tibia	–	513.5	15.7	136	0	–	–
7218	radius/tibia	29.6	504.2	16.5	172	6	0.4	–
7130	tibia	28.7	539.7	16.3	167	0	–	–

“MW”  =  Midshaft Width; “TBA”  =  Total Bone Area; “Vasc”  =  vascularization; “AvPOD”  =  Average Primary Osteon Diameter; “K”  =  ratio between the internal and external diameter of the bone (cortical thickness) [38] measured for limb bones with a preserved midshaft region; “C”  =  bone compactness [40].

All elements were measured and photographed prior to thin sectioning. Because the mid-diaphysis of limb bones experiences the least amount of secondary remodelling and thus provides the most complete information about life history ([28]–[32]), elements were thin sectioned in this region wherever possible (section locations are noted for each element below). Thin sectioning was conducted at the National Museum, Bloemfontein, following procedures outlined by Chinsamy & Raath [33] with minor modifications. The bone microstructure was viewed and photographed using a Nikon Eclipse 50i Polarizing microscope and DS-Fi1 digital camera. Bone histology terminology follows that of Francillon-Vieillot et al. [31] and Reid [34].

The relative vascular area or cortical vascularity (also known as cortical porosity or vascular density) in the primary cortex was quantified using the Nikon software NIS elements D. Because the organization and density of the vascular canals in cortical bone is closely related to bone apposition rate (e.g. [35]–[36]), this method allows the vascular density to be compared quantitatively, not only between different elements, but also between different taxa. This technique produces the maximum possible vascularization value for each element because the channels would have included lymph and nerves as well as blood vessels in life ([37]). Cortical vascularity was calculated by arbitrarily selecting ten standardized quadrants from the midcortex of each section (thus excluding areas of secondary remodelling). The total canal area was calculated and divided by the total cortical area and then multiplied by 100 to obtain a percentage. Mean primary osteon diameter also reflects bone apposition rates and is generally highest in highly woven fibro-lamellar bone ([36]). Mean primary osteon diameter was calculated (in microns) by measuring the transverse lengths of primary osteons in the cortex.

The thickness of the cortex and the overall compactness of the bone were measured in order to assess the robustness of the bones and to make inferences regarding lifestyle. The midshaft cortical thickness (k, expressed from 0 to 1 where cortical thickness is thickest when k tends towards 0, [38]) was recorded for all limb bones by measuring the ratio between the inner diameter and outer diameter of the bone. A bone compactness profile (defined as “the ratio between the surface occupied by bone tissues and the total bone surface”, [39]: 337) was also obtained from the midshaft of each sectioned limb bone using the computer program Bone Profiler for Windows [40]. This profile represents the ratio between the mineralized tissue surface and the total cross-sectional surface [39]–[42]. Compactness is higher towards a value of 1.

### Geological Setting

Welman et al. [43] reported the presence of *Cynognathus* Assemblage Zone (AZ) fossils from the lower Burgersdorp Formation of the northeastern and northwestern Free State. These authors also provided an initial faunal list that included the archosauriform *Erythrosuchus*, but this material is here identified as the new species of *Garjainia* described herein. Subsequent to the work of Welman et al. [43], the northern Burgersdorp Formation exposures have been the focus of various sedimentological and stratigraphic studies [21]–[22], [44]–[47].

**Figure 2:**
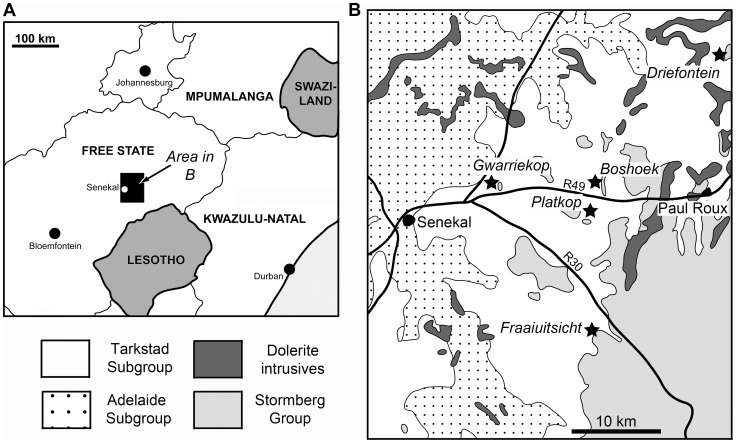
Localities yielding fossils of *Garjainia madiba* sp. nov. Localities indicated by stars in part B. Only the main (BP material) localities are indicated, other (NM material) localities lie a short distance to the east.

Exposures of the lower Burgersdorp Formation in the northern Free State occur as discontinuous outcrops throughout, and to the east of the study area (Fig. 2). Four main localities have been investigated, with supplementary data from three additional localities, all these lying in Thabo Mofutsanyana district municipality. The main localities are located on the farms Driefontein 11, Gwarriekop 330, Boshoek and Fraaiuitsicht/Bosrand 12. Driefontein 11 is of particular importance because it is taxonomically the most diverse site within the northern Burgersdorp Formation exposures, and in fact within the entire *Cynognathus* AZ. The majority of the material of the new species reported here was collected from Driefontein 11, but one of the most complete specimens (BP/1/5525) was recovered from Gwarriekop 330 (also referred to as Ghwarriekop or Guarriekop 330), one of the sites listed by Welman et al. [43].

**Figure 3:**
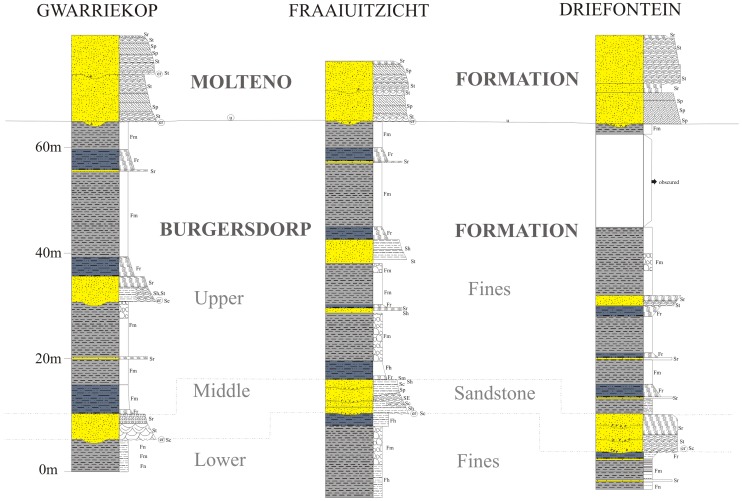
Stratigraphic sections of localities yielding *Garjainia madiba* sp. nov. Facies codes follow those of [106]. er  =  erosional contact; F (Fines - siltstones and mudstones): Fh  =  horizontally laminated, Fm  =  massive, Fr  =  ripple cross-stratified; S (Sandstones): Se  =  intraformational erosional scour, Sh  =  horizontally stratified, St  =  trough cross-stratified, Sp  =  planar cross-stratified, Sl  =  low angle planar stratified, Sr  =  ripple cross-stratified.

**Figure 4:**
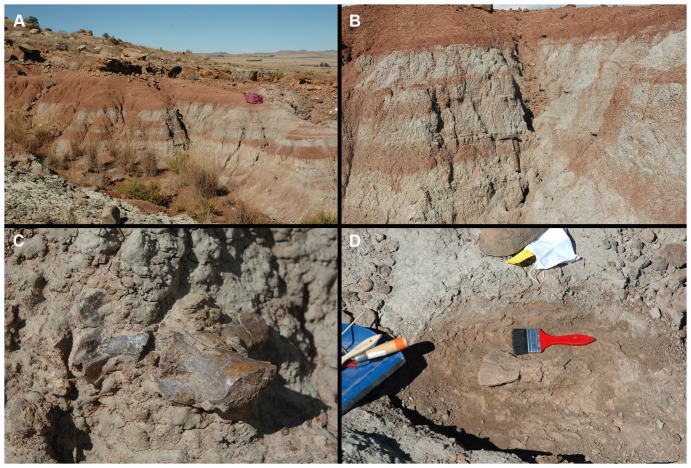
Photographs of specimens of *Garjainia* in field and outcrop. Holotype locality (A, B), with the position of the holotype specimen marked by a hammer. Part of holotype specimen in the field (C). Scapula of *Garjainia* in situ at the top of the holotype section (D).

Stratigraphic sections for Driefontein, Gwarriekop, and Fraauitsicht are presented in Fig. 3. All three localities preserve a similar stratigraphy that can be divided into a lower, horizontally-laminated to massive, fines- (siltstones and mudstones) dominated unit, disconformably overlain by a more sandstone-rich middle succession, and an upper, fines-dominated unit. The lower, mudrock dominated unit is between 5–15 m thick, and is composed of dark reddish brown (10R 3/4) fines that are generally laminated to massive in nature. Although no articulated vertebrate fossil material has been discovered, this unit generally preserves the most complete and best-preserved specimens, usually as isolated elements (Fig. 4). It is interpreted as a lacustrine succession, with the total disarticulation of the specimens caused by bottom current activity. The holotype (BP/1/5760) was discovered at the top of this succession (Fig. 4).

The middle, sandstone unit may be up to 10 m thick, and is best developed on the farm Fraaiutsicht (Fig. 3c). The base of the unit (and any basal unit within the succession) may evidence erosional scour and be overlain by a clay pebble, reworked burrow casts, bone and coprolite lag accumulation. The depositional sequence is dominated by light grey (5Y 7/1), fine- to medium-grained, trough cross-stratified and ripple cross-stratified sandstones. The taphonomy of the material from the middle sandstone horizon is distinctly different from that of the lower laminated fines unit, with the bulk of the fossils being fragmentary and showing evidence of mixed taphonomic histories and abrasion. This unit is interpreted as representing a fairly high sinuosity, low energy channel depositional environment.

The upper part of the sequence is formed by a thick (up to 40 m) succession of dark reddish-brown (10R 3/4) fines, and intercalated thick (up to 8m) thin (<1 m) sandstones. These become more greyish brown (5YR 3/2) towards the top of the sequence. The mudrock units show horizontal lamination, as well as evidence of stacking of weakly developed calcic palaeosols. These palaeosols show only incipient vertic structure, with mudcracks filled by fine blue siltstone. The upper, mudrock dominated succession preserves only isolated bone elements that often have thick encrustations of haematite and calcite. The overall thickness of the succession coupled with the lack of well-defined and in places truncated palaeosols is indicative of fairly rapid overbank floodplain aggradation, punctuated by periods of incipient soil development, with a lower water table than for the underlying floodbasin fines sequence. At all the localities the upper sequence is unconformably overlain by the Late Triassic Molteno Formation [45], which comprises two medium- to coarse-grained sandstone units (Fig. 3).

The new species is known almost exclusively from sedimentary units likely deposited in fluviolacustrine environments. In the lower fines unit the associated vertebrate assemblage includes only temnospondyl amphibians and actinopterygian, sarcopterygian and chondrichthyan fish. Fossils of terrestrial vertebrates are known from the overlying middle sandstone and upper fines units and include small cynodonts, procolophonids, theracephalians and *Palacrodon* [43], [46], [48], [49].

## Results

### Systematic Palaeontology

Diapsida Osborn, 1903 [50]

Archosauromorpha Huene, 1946 [51] *sensu* [1]

Archosauriformes Gauthier, 1986 [13]

Erythrosuchidae Watson, 1917 [12] *sensu* [52]

*Garjainia* Ochev, 1958 [16]

#### Type species

*Garjainia prima* Ochev, 1958 [16] by original monotypy. Yarenskian ( =  Yarengian) Gorizont of Orenburg Province, European Russia (‘*Parotosuchus* fauna’, latest Early Triassic, late Olenekian: [53]).

#### Revised Diagnosis

Erythrosuchid archosauriforms differing from other confirmed erythrosuchid genera (*Erythrosuchus*, *Shansisuchus*) in having the following combination of features (*probably plesiomorphic for Archosauriformes and Erythrosuchidae; **probably apomorphic for *Garjainia* within Erythrosuchidae): *lack of premaxilla-maxilla peg-socket joint (present in *Erythrosuchus* and *Shansisuchus*); *large and prominent ventral ramus of the opisthotic (relatively much smaller ventral ramus present in *Erythrosuchus* and *Shansisuchus*); *single antorbital fenestra between orbit and external naris (accessory antorbital opening present in *Shansisuchus*); *quadratojugal makes substantial contribution to ventral part of posterior border of lateral temporal fenestra (contribution of quadratojugal very small or absent in *Erythrosuchus* and *Shansisuchus*); **posteriorly divergent medial borders of exoccipitals on floor of foramen magnum (subparallel in *Erythrosuchus*, *Shansisuchus* and most outgroups, but not e.g., *Fugusuchus hejiapensis*: [54]); ** clearly differentiated ambiens process on pubis (weakly differentiated in *Erythrosuchus*, *Shansisuchus* and outgroups); **tight and substantial articulation between posterior face of distal end of the ventral ramus of the opsithotic and basioccipital (simpler, looser, briefer contact in *Erythrosuchus* and *Shansisuchus*); **anteroventrally-posterodorsally oriented antorbital fenestra widely separated from external naris (not known in the new species; long axis of fenestra more horizontal in *Erythrosuchus* and outgroups). Sources of uncertainty here include lack of knowledge about some features in some taxa, lack of inclusion of some taxa in phylogenetic analyses, and lack of phylogenetic robustness for some relationships.

Several additional poorly known taxa might also be erythrosuchids [10] and *Garjainia* differs from them in various features. For example, *Garjainia* differs from *Chalishevia cothurnata* Ochev, 1980 [55] in having a single antorbital fenestra (*versus* two) between the orbit and external naris, in lacking an extensive antorbital fossa on the maxilla, and in having more maxillary teeth (at least 13 *versus* 11); from *Youngosuchus sinensis* (Young, 1973 [56]) in having a more downturned premaxilla with more than four (*versus* fewer than four) teeth, and in having a broad and well-developed posterior premaxillary process that excludes the maxilla from the external naris; from *Guchengosuchus shiguaiensis* Peng, 1991 [57] in having a single antorbital fenestra (*versus* two), a prominent lateral ridge on the ascending process of the maxilla (*versus* absent), relatively shorter cervical vertebral centra, and in having cervical neural spines that are not transversely expanded and rugose distally; from *Uralosaurus magnus* (Ochev, 1980 [55]) in having substantially more than 10 dentary teeth.

#### Content

*G. prima* Ochev, 1958 [16] and *G. madiba* sp. nov.

#### Remarks

The species of *Garjainia* are very similar in most respects. It is likely that more character states will emerge as being clearly diagnostic for the genus as other early archosauriforms become better known. For a morphological diagnosis of Erythrosuchidae see Discussion.

Garjainia madiba sp. nov.

urn: lsid: zoobank.org: act: 10D4C8F1-E39B-44B8-924B-71EB0A451A63

“Primitive erythrosuchid thecodonts”: Hancox et al. ([21]: 143)

“*Garjainia*”: Hancox ([22]: 1299)

“New taxon…very similar to the genus *Garjainia*”: Hancox & Rubidge ([58]: 566)

“New form, similar to the genus *Garjainia*”: Rubidge ([59]: 139, 156)

“*Garjainia*-like erythrosuchids”: Rubidge ([59]: 149)

“*Garjainia*”: Abdala et al. ([60]: 384)

“carnivorous archosaurian reptile”: Cisneros et al. ([61]: 1)

“basal archosaurian reptile”: Cisneros et al. ([61]: 1, 4; fig. 1)

“carnivorous reptile”: Cisneros et al. ([61]: 1, 4)

“*Garjainia*?”: Smith et al. ([62]: table 2.5)

“cf. *Garjainia*”: Smith et al. ([62]: 50)

“Undescribed erythrosuchid”: Ezcurra et al. ([10]: 15)

“Undescribed, *Garjainia*-like taxon”: Ezcurra et al. ([10]: table 1)

“new species of *Garjainia*”: Butler et al. ([63]: 98)

#### Holotype

BP/1/5760, collected by P.J. Hancox in 1999 and 2000. Right postorbital, right postfrontal and partial frontal in articulation; ventral end of left postorbital; left jugal; right paroccipital process with partial supraoccipital and parts of right prootic in articulation; partial rib; at least four unidentified fragments, some of which likely represent partial skull elements. The holotype elements were found within 50 cm diameter of each other and some pieces can be articulated with each other (i.e., right postorbital + postfrontal/frontal; left postorbital fragment + jugal). A partial left premaxilla (BP/1/5760a) was found close to (ca. 20 cm away from) the holotype material but was less obviously associated and is not considered part of the holotype, but as a paratype. An isolated cervical vertebra from the same horizon and locality (BP/1/5760b) has been treated in the same way. The number and size of the holotype elements are consistent with their having come from a single individual.

#### Paratypes

Approximately 80 specimens from various localities. See Text S1 for details.

#### Referred material

Sixteen specimens from various localities. See Text S1 for details.

#### Diagnosis

Differs from its only congener (*G. prima*) in having: (1) large bosses on the lateral surfaces of the jugal and postorbital, these being unknown in any other erythrosuchid or non-archosaurian archosauriform and therefore being autapomorphic for the species. The dorsal end of the quadratojugal is also somewhat thickened (based on a single example). (2) Higher tooth counts for the premaxilla (six *versus* five) and maxilla (likely more than 14 *versus* 13 or 14); (3) A longer postacetabular process of the dorsal blade of the ilium (approximately as long as the acetabular portion of the ilium *versus* clearly shorter than the acetabular portion of the ilium).

#### Type locality

Farm Driefontein 11, approximately 36 km NE of Senekal and 14 km N of Paul Roux, Thabo Mofutsanyane district municipality, Free State, South Africa (see Fig. 2). The locality has been entered into the Paleobiology Database and is locality 98617.

#### Type horizon

Burgersdorp Formation (Beaufort Group), *Cynognathus* Assemblage Zone Subzone A (late Early Triassic, late Olenekian: [22]).

#### Distribution

Known only from the *Cynognathus* Assemblage Zone Subzone A of the Burgersdorp Formation, South Africa. Known from several localities to the east and northeast of Senekal, Free State (see Fig. 2; Text S1).

#### Etymology

Named in honour of Nelson Mandela (1918–2013), the first fully representatively democratically elected president of South Africa (1994–1999). Mr Mandela was known affectionately as “Madiba”. The specific epithet is considered a noun in apposition.

#### Remarks

Even if *Garjainia triplicostata* (Huene, 1960 [23]) is considered a valid species distinct from *G. prima* (*contra* [9]), the diagnosis for the new species described here stands. The most obvious difference between the new species and its congener(s) is the presence of large (and autapomorphic) bony bosses in the cheek region of *G. madiba*. The approximately hemispherical boss on the jugal is known from several examples and differs from the lower sub-longitudinal ridge in the corresponding position of all known (five) jugals of the Russian species (PIN 2394/5, 951/19 and 951/23). The jugal of the holotype of *G. madiba* is approximately the same length as that of the holotype of *G. prima*, the former has a very thick boss and the latter only a low ridge. The boss on the postorbital of *G. madiba* is also much more prominent and hemispherical than the relatively low brow on the corresponding element of *G. prima*. The longer postacetabular process of the ilium in *G. madiba* and typically higher premaxillary tooth count are other clear differences between the species that are based on multiple examples. A small difference in the estimated number of maxillary teeth will likely require reassessment once larger samples of all species are obtained. The same is true of several other minor differences noted below in the description of the braincase of *G. madiba*.

Much of the material that we designate as paratypes of *G. madiba* consists of isolated and/or fragmentary remains that do not necessarily include elements that overlap with those of the holotype. Identification of these specimens as *G. madiba* is based upon their very close similarity to *G. prima* (and other erythrosuchids such as *Erythrosuchus africanus* and *Shansisuchus shansisuchus*), their similar (generally large) size, and the assumption that a single erythrosuchid species is present within this stratigraphic horizon in South Africa (which is supported by the lack of substantial variation within our sample). As such, our identifications represent hypotheses that will be tested by future discovery of more complete and associated material of *G. madiba*, and we choose to describe the holotype specimen separately from the rest of the skeletal material. Some material that is especially fragmentary and/or for which we are less confident in making our identification are considered referred specimens rather than paratypes. These have not been figured or used in the description of the new species here (except for bone histology), and are listed separately in Text S1.

Several isolated (generally smaller) archosauromorph/archosauriform elements found in the same horizon and at the same localities as *G. madiba* are not referred here to the new species because they do not closely resemble known elements of erythrosuchids and/or belong demonstrably to different archosauromorph taxa. For example, an incomplete, small right ilium (BP/1/6232p) lacks a strongly developed supraacetabular rim and post-acetabular extension of the articulation surface for the ischium, and is relatively short anteroposteriorly and deep dorsoventrally compared to ilia of *G. madiba*. It also has a much shorter preacetabular process of the dorsal blade than seen in *G. prima* and other erythrosuchids (unknown in *G. madiba*). Two humeri (BP/1/6232s, BP/1/5661) are much more slender than those of *G. madiba* and have much shorter deltopectoral crests.

### Description of Holotype Specimen

**Figure 5:**
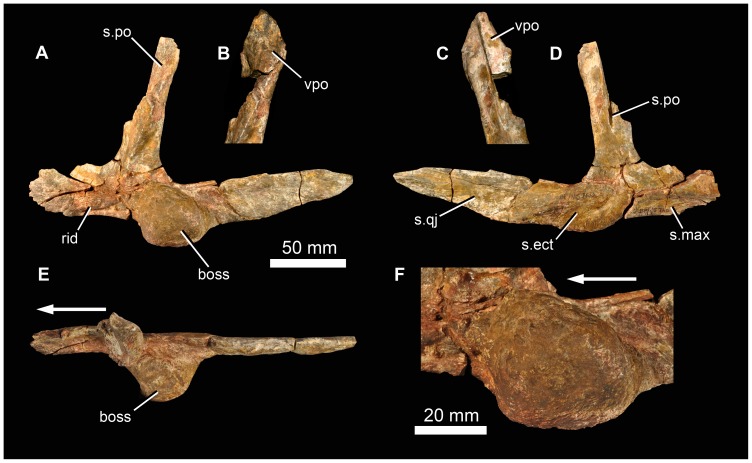
Jugal and part of postorbital of holotype (BP/1/5760) of *Garjainia madiba* sp. nov. A, D–F, left jugal in lateral (A), medial (D), and dorsal (E) views, with lateral close-up of jugal boss (F). B–C, dorsal (postorbital) process of left jugal in articulation with fragment of ventral process of postorbital in lateral (B) and medial (C) views. Abbreviations: boss, jugal boss; rid, ridge; s.ect, surface for articulation with ectopterygoid; s.max, surface for articulation with maxilla; s.po, surface for articulation with postorbital; s.qj, surface for articulation with quadratojugal; vpo, ventral process of postorbital. Arrows point to anterior of element.

The strongly triradiate left jugal of BP/1/5760 is almost complete (Fig. 5). Its most notable feature is a massive, almost spherical, laterally projecting boss on the lateral surface of the anterior end of the posterior process, just posterior to the base of the postorbital process. This boss has a rugose surface texture. Medially, the anterior process of the jugal bears a subhorizontal ridge, and the anteroventral edge has a slot-like facet for articulation with the maxilla (Fig. 5: s.max). A low anteriorly-extending ridge is also present on the lateral surface of this process, close to its ventral margin (Fig. 5: rid). The medial surface of the posterior process bears a long facet for the lateral surface of the quadratojugal (Fig. 5: s.qj). This facet terminates just posterior to the boss. Within the most posterior part of the facet there is a low anteroposteriorly-extending ridge. A shallow concavity with a slightly roughened ventral edge lies medial to the boss (Fig. 5: s.ect), this likely articulated with the head of the ectopterygoid, which would have been close to, or might have contacted, the posterior end of the maxilla, as in *G. prima* ([64]: fig. 1). The dorsal process of the jugal bears a large facet for the descending process of the postorbital (Fig. 5: s.po). Dorsally, this facet lies on the lateral (and anterior) face of the jugal, but further ventrally it is much more on the anterior margin, and eventually is seen as a vertical groove on the medial surface of the base of the dorsal process, close to the lower edge of the border of the orbit.

**Figure 6:**
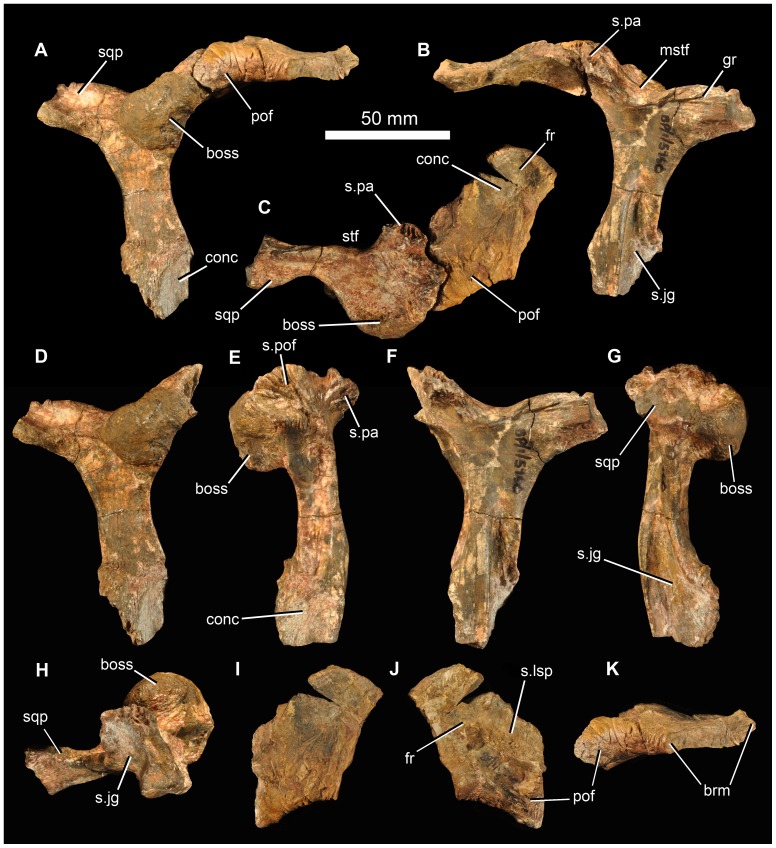
Frontal/postfrontal/postorbital of holotype (BP/1/5760) of *Garjainia madiba* sp. nov. A–C, right postorbital in articulation with right postfrontal and partial right frontal in lateral (A), medial (B) and dorsal (C) views. D–H, right postorbital in lateral (D), anterior (E), medial (F), posterior (G) and ventral (H) views. I–K, right postfrontal and partial frontal in dorsal (I), ventral (J) and lateral (K) views. Abbreviations: boss, postorbital boss; brm, broken margin; fr, frontal; gr, groove; mstf, margin of supratemporal fenestra; pof, postfrontal; s.jg, surface for jugal; s.lsp, concave surface for articulation with laterosphenoid; s.pa, surface for parietal; s.pof, surface for postfrontal; sqp, squamosal process of postorbital; stf, supratemporal fenestra.

Fragments of the right frontal and postfrontal are in firm articulation (Fig. 6), and both can be articulated with the right postorbital of the same specimen. The contact between the frontal and the postfrontal is difficult to determine, but the latter element appears to be small and approximately triangular in dorsal view, and forms a thickened and slightly rugose posterodorsal border of the orbit (Fig. 6: pof). The frontal fragment resembles the homologous region in *G. prima* and *E. africanus*. It has a shallow concavity on the dorsal surface, and the ventral surface is marked by a distinct medially located concavity (Fig. 6C, J) that is bounded laterally by a sharp rim, continuous with a rim on the postorbital. This concavity marks the articular surface for the laterosphenoid.

The right postorbital of BP/1/5760 is well preserved (Fig. 6). The most notable feature is a greatly thickened, hemispherical dorsolateral boss, positioned at the posterodorsal corner of the orbit. This boss has a rugose surface texture and its dorsal surface (adjacent to the supratemporal fenestra) is concave. The boss greatly overhangs the descending process of the postorbital. This process is L-shaped in cross-section, with a shorter anterior margin (which is marked by a concave, dorsoventrally extending furrow) and a broader lateral margin (which is anteroposteriorly concave). Ventrally, the descending process broadens in lateral view, and is concave on the anterolateral surface of its distal end (Fig. 6A, E: conc). The facet for the dorsal process of the jugal extends up the descending process of the postorbital for more than half of its length (Fig. 6B: s.jg). The posterior process for the squamosal is incomplete, but bears grooves and low ridges on its medial surface close to its base. The anteromedial process for the parietal is short (Fig. 6E: s.pa), and the postorbital formed the anterolateral corner of the margin of the supratemporal fenestra. The left postorbital of the holotype is represented only by a fragment of the ventral end that can be articulated with the dorsal process of the left jugal (Fig. 5B, C).

**Figure 7:**
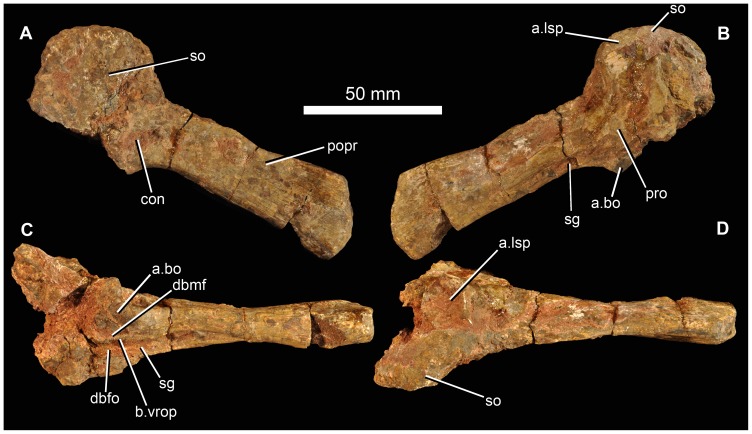
Partial braincase of holotype (BP/1/5760) of *Garjainia madiba* sp. nov. A–D, right paroccipital process in articulation with supraoccipital and right prootic in posterodorsal (A), anteromedial (B), ventral and slightly medial (C) and dorsal and slightly lateral (D) views. Abbreviations: a.bo, articular surface for basioccipital; a.lsp, articular surface for the laterosphenoid; b.vrop, broken ventral process of opisthotic; con, concavity; dbmf, dorsal border of metotic foramen; dbfo, dorsal border of fenestra ovalis; popr, paroccipital process; pro, prootic; sg, stapedial groove; so, supraoccipital.

The right paroccipital process of BP/1/5760 (Fig. 7) is almost complete though incompletely prepared. It is preserved in articulation with the supraoccipital and parts of the right prootic but the sutures among these elements cannot be readily traced in the current state of preparation. The paroccipital process is slightly expanded dorsoventrally at its distal end in posteromedial view, with fairly straight dorsal and ventral edges, the latter being slightly longer. The distal end of the process in posteromedial and anterolateral views is curved with its apex slightly ventral to the dorsoventral midpoint. Distally the process is narrower at its dorsal edge than ventrally, and the distal surface is slightly concave. The stapedial groove extends out onto the ventral edge of the anterolateral face of the process (Fig. 7B, C: sg). The ventral ramus of the opisthotic has mostly broken away ventrally. Posteromedially the proximal base of the paroccipital process has a shallow concavity (Fig. 7A: con) as in *G. prima* [54] and *E. africanus* [19]. The dorsal surface of the prootic has a concave articular facet for articulation with the laterosphenoid (Fig. 7B, D: a.lsp). The supraoccipital (Fig. 7A: so) resembles that of *G. prima* more than *E. africanus* in its greater breadth relative to the length of the paroccipital process. It has a flat to slightly concave posterior surface and its dorsal margin is convex in posterior view.

**Figure 8:**
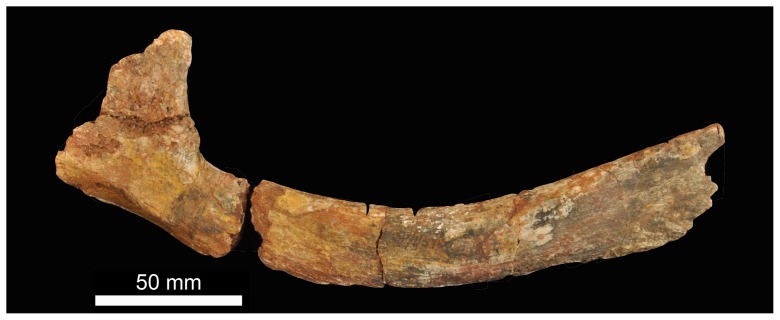
Partial braincase of holotype (BP/1/5760) of *Garjainia madiba* sp. nov.

BP/1/5760 includes a fragmentary probable dorsal rib (Fig. 8) on the basis that it lacks any indication of being a cervical, sacral or caudal rib (which have a more distinctive morphology in early archosauriforms). It is curved along its length, with the shaft of the rib being composed of an inner rod-like part and outer flange that tapers distally.

### Additional Information from Paratypes

#### Premaxilla

**Figure 9:**
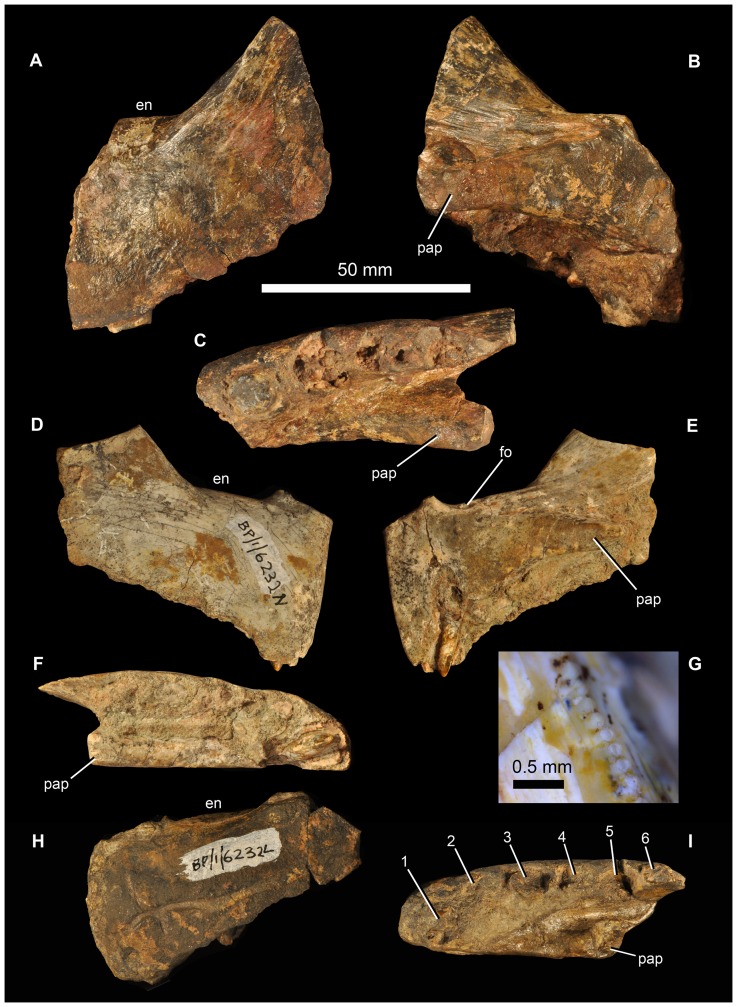
Premaxillae of *Garjainia madiba* sp. nov. **A–C**, BP/1/5760a, partial left premaxilla in lateral (A), medial (B) and ventral (C) views. D–G, BP/1/6232n, partial right premaxilla in lateral (D), medial (E) and ventral (F) views, and close up of distal margin of first premaxillary tooth (G). H–I, BP/1/6232l, partial left premaxilla in lateral (H) and ventral (I) views. Abbreviations: en, border of external naris; fo, ethmoidal foramen; pap, palatal process. Numbers in part I indicate tooth alveoli.

**Figure 10:**
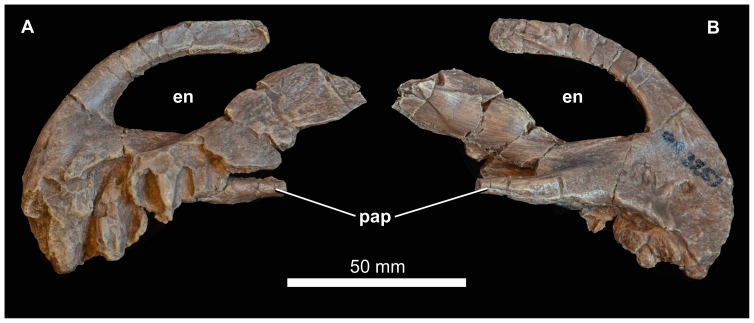
Premaxilla of *Garjainia madiba* sp. nov. Synthetic cast of NMQR 3257, left premaxilla in lateral (A) and medial (B) views. Abbreviations: en, border of external naris; pap, palatal process.

There are no complete premaxillae, but the morphology of the preserved portions (BP/1/5525, two partial right premaxillae; BP/1/5760a, partial left premaxilla; BP/1/6232n, relatively complete right premaxilla; BP/1/6232l, partial left premaxilla; BP/1/7138, partial right premaxilla; NMQR 3257, left premaxilla) (Figs. 9, 10) is very similar to that of *G. prima*. Although the posterior part of the ventral margin of the premaxilla (alveolar margin) is slightly damaged in most examples, the main body of the premaxilla appears to be subquadrangular in lateral view, with the ventral border of the naris being approximately parallel to the alveolar margin. In lateral view, the anterior margin of the premaxilla is vertically or even anterodorsally orientated, rather than sloping slightly posterodorsally as in *E. africanus* [20]. The posterodorsal process has a broad base behind the naris. The posterior edge of the premaxilla is thin (BP/1/6232n) and the preserved portions lack any indications of the peg seen in *E. africanus* [20], *S. shansisuchus* ([17]: fig. 8b) or *S. kuyeheensis* [65]. In lacking a border for a second antorbital fenestra the premaxilla of *G. madiba* more closely resembles that of *G. prima* [9] and *E. africanus* [20] than *S. shansisuchus* [17]. In lateral view the long axis of the slender prenarial (nasal) process of the premaxilla of *G. madiba* is almost vertical anteriorly and curves to become almost horizontal dorsally and posteriorly (NMQR 3257). The postnarial process (best preserved in NMQR 3257, Fig. 10) is strongly transversely compressed. As in *E. africanus* [20], there is a foramen immediately behind the medial surface of the base of the prenarial process (Fig. 9E: fo), and a cluster of foramina are present on the anterolateral surface of the premaxilla, close to the alveolar margin (BP/1/6232n). Gower ([20]: 15, 81) variably identified the former foramen as for the ethmoidal nerve and ethmoidal artery, and based on comparisons with extant diapsids (e.g. [66], [67]: 17) it probably carried a branch of the ophthalmic nerve and possibly associated blood vessels.

The lateral surface of the premaxilla of BP/1/6232L (Fig. 9H) bears a groove subparallel to the dental margin. This likely held neurovascular tissue in life, and is seen also in *G. prima* and in at least some specimens of *E. africanus* (BP/1/5207). The groove is not clear in all the *G. madiba* specimens, possibly resulting from individual variation as well as differences in preservation (Fig. 9).

The premaxilla of *G. madiba* clearly formed a downturned snout tip, with a broad notch in the ventral border of the jaw between premaxilla and maxilla, as seen in *E. africanus* [20] and the holotype of *G. prima* [16], [68]. The downturning of the premaxilla appears to be more strongly developed in *Garjainia* spp. than in other erythrosuchids. Much of the medial surface of the main body of each premaxilla of *G. madiba* forms a facet for its antimere. This facet tapers posteriorly onto the element's palatal process (Figs. 9, 10: pap). As a consequence of the downturned snout, the posterior tip of the palatal process approaches (and, when complete, possibly reached or extended beyond) the posteroventral corner of the premaxilla in lateral and medial views (Figs. 9, 10). There are no complete premaxillae known for *G. madiba* and the alveolar border is eroded to some degree in all specimens, but the downturning of the snout and premaxilla is less strong than in proterosuchids (see [69]). For example, the pre- and postnarial processes are nearly complete in NMQR 3257 and they do not converge to completely close the posterior end of the naris, and the palatal process and alveolar margin are at a shallower angle to each other (e.g., BP/1/5760a and BP/1/6232n) than in for example, *Proterosuchus fergusi*.

Where the ventral edge of the premaxilla is adequately preserved to make a complete count there are six alveoli (possibly five in NMQR 3257). The slightly smaller right premaxilla of BP/1/5525 might have held fewer than six teeth but the anterior edge is too incomplete to make a complete count with confidence. Five teeth occur in the documented material of *E. africanus* [20], *G. prima* ([16], [68]; pers. obs.), and *S. shansisuchus* (DJG, pers. obs.; [14]; [17]: 127 *contra* table 11). At first glance, NMQR 3257 (Fig. 10) appears to differ notably from the other (Fig. 9) *G. madiba* premaxillae and from the premaxilla of *G. prima*, and bears closer resemblance to the premaxilla of *Sarmatosuchus otschevi*. However, the alveolar margin of NMQR 3257 is substantially eroded and incomplete, especially posteriorly, and much of the lateral surface of the premaxillary body (laterally covering the alveoli) has also been lost. The specimen is also substantially smaller than the known premaxillae of *G. prima* and most of the known *G. madiba* premaxillae, and when this, and the erosion of NMQR 3257, are taken into account there is little difference in overall form.

#### Maxilla

**Figure 11:**
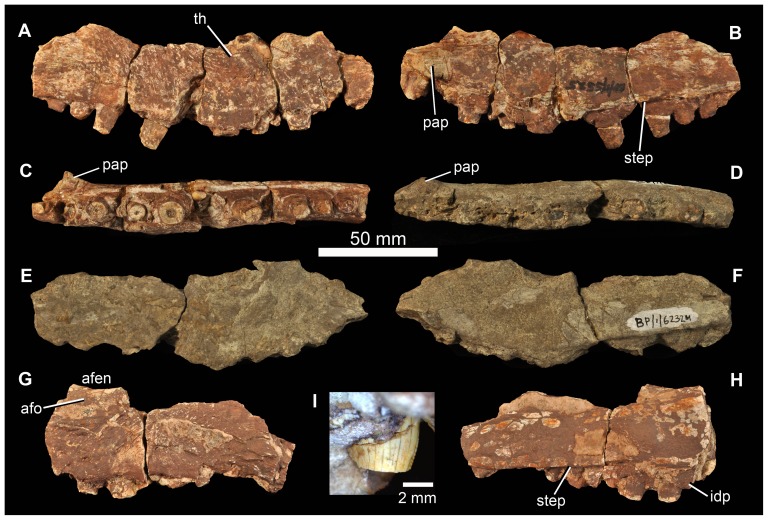
Maxillae of *Garjainia madiba* sp. nov. A–C, BP/1/5525, right maxilla in lateral (A), medial (B) and ventral (C) views. D–F, BP/1/6232m, right maxilla in ventral (D), lateral (E) and medial (F) views. G–H, BP/1/7138, partial left maxilla in lateral (G) and medial (H) views. I, close-up of replacement tooth of right maxilla of BP/1/5525. Abbreviations: afen, antorbital fenestra; afo, antorbital fossa; idp, interdental plate; pap, palatal process; step, step on medial surface above alveolar margin; th, curved thickening that was likely continuous with ridge on the ascending process.

All preserved examples (Fig. 11) are incomplete (BP/1/5525, right maxilla, possible fragment of second right maxilla; BP/1/6232m, right maxilla; BP/1/7138, haematite-encrusted left maxilla). The ventral, tooth-bearing margin is markedly convex along most of its length (except posteriorly) in lateral view, and holds at least 13 teeth but, based on comparison of the preserved fragments with maxillae of other erythrosuchids, especially *G. prima*, probably not more than 18. The anterior ascending process (forming the anterodorsal border of the antorbital fenestra) is missing in all specimens, but its base lies above the approximately fourth and fifth preserved alveoli in BP/1/5525. The border of the antorbital fenestra is damaged in all specimens, although there is a slightly curved thickening at the anterior end of the ventral margin of the fenestra in BP/1/5525 (Fig. 11A: th) that likely was continuous with a ridge on the ascending process. A small part of the antorbital fossa appears to be preserved in BP/1/7138 (Fig. 11G: afo). The palatal process is restricted to the anterior of the maxilla, anterior to the base of the ascending process (Fig. 11: pap). The medial surface bears a low step running subparallel to the ventral (alveolar) margin of the maxilla (Fig. 11B, H: step). Somewhat irregular and not well-preserved interdental plates are present, particularly in BP/1/5525 and BP/1/7138 (Fig. 11H: idp). The posterior part of the maxilla has a thick ventral and medial portion, and a thin sheet-like dorsal and lateral portion (BP/1/7138, Fig. 11H) beneath the antorbital fenestra, as also described for *E. africanus* ([20]: 17). Because all of the maxillae are damaged it is not possible to determine if they expanded in height posteriorly, as in *G. prima*. The lateral surface of the maxilla has a slightly rugose surface texture below the antorbital fenestra.

#### Nasal and lacrimal

No nasals or lacrimals have been identified among the preserved material.

#### Prefrontal

**Figure 12:**
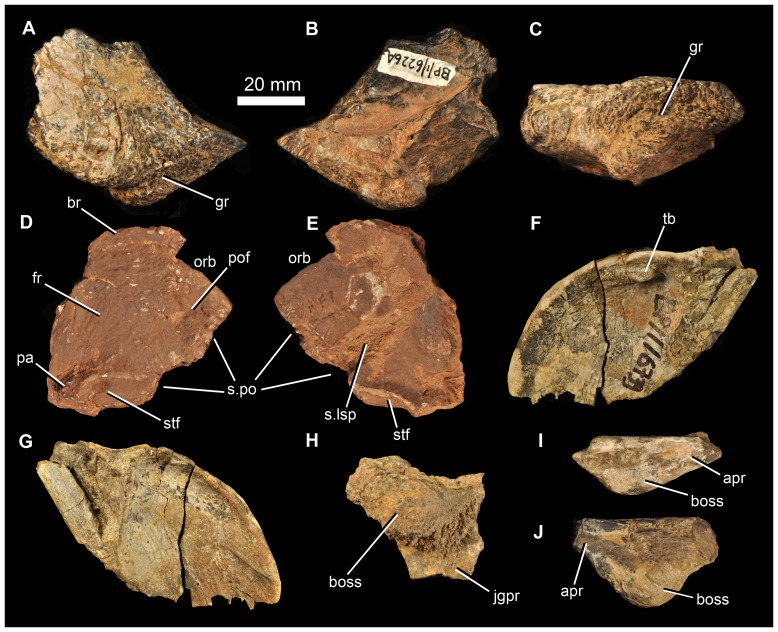
Various cranial elements of *Garjainia madiba* sp. nov. A–C, BP/1/6226a, partial right prefrontal in dorsal (A), ventral (B) and lateral (C) views. D–E, BP/1/5525, skull roof fragment comprising partial right frontal, right postfrontal, and anterior end of right parietal in dorsal (D) and ventral (E) views. F–G, BP/1/6739, occipital flange of left parietal in posterior (F) and anterior (G) views. H, BP/1/6232ag, partial left postorbital in lateral view. I, BP/1/6232h, partial right jugal in dorsal view. J, BP/1/6232i, partial left jugal in dorsal view. Abbreviations: apr, anterior process of jugal; boss, jugal or postorbital boss; br, broken margin; fr, frontal; gr, groove; jgpr, jugal process of postorbital; pa, parietal; pof, postfrontal; orb, orbital margin; s.lsp, surface for articulation with laterosphenoid; s.po, sutural surface for postorbital; stf, supratemporal fossa; tb, tubercle.

A small piece of the roofing part of a right prefrontal is preserved (BP/1/6226a: Fig. 12A–C) and is similar to that of *G. prima* and *E. africanus*. The anterior part of the bone has broken away. The bone has a rugose external surface texture, and its dorsal surface is gently concave medially, towards the contact with the frontal. The roof slightly overhangs the incompletely preserved descending process of the prefrontal. The lateral edge of the prefrontal is thickened dorsoventrally, and medially the element is thinner (excavated ventrally) adjacent to the contact with the frontal. The lateral surface bears a broad, primarily subhorizontal, shallow groove (Fig. 12C: gr), which trends anterodorsal-posteroventral adjacent to the orbital margin. A similar groove is present in *E. africanus* [20]. The prefrontal formed the anterodorsal corner of the orbital rim.

#### Frontal

BP/1/5525 includes a skull roof fragment (Fig. 12D–E) that includes a substantial part of the right frontal, most of the right postfrontal, as well as the anterolateral part of the right parietal. The frontal is broken anteriorly, and damaged along its medial margin. It appears to have formed at least a small portion of the orbital margin. Ventrally, a well-developed transverse ridge forms the anterior margin of a concavity on the frontal and parietal that (based on comparison with *G. prima*) articulated with the laterosphenoid. Curved ridges on the dorsal and ventral margins of the parietal represent the margins of the right supratemporal fossa.

#### Parietal

Beyond the fragment of parietal present in the partial skull roof of BP/1/5525, only a single, partial parietal is preserved (BP/1/6739), representing the thin occipital flange of the left side (Fig. 12F–G). The posterior surface of the flange is gently concave, and is thickened at its dorsal margin (such that the flange has a somewhat T-shaped cross section). The preserved piece bears a small tubercle dorsomedially on the posterior surface, present also in *E. africanus* [20]. The occipital flange of the parietal of *G. madiba* has a tightly curved dorsolateral margin in posteromedial/anterolateral view. In this respect it is this more similar to *G. prima* as figured by Ochev [68] than *E. africanus* as figured by Gower [20] but we are not convinced that this is a generic difference because of variation in *E. africanus* ([20]: fig. 9) and because the shape of this flange varies depending on the view (see for example the more posteroventral view presented by [54]: fig. 1).

#### Postorbital

The best example is preserved as part of the holotype. Several additionally fragmentary postorbitals (BP/1/6232ad, partial left postorbital; BP/1/6232ag, Fig. 12H, smaller partial left postorbital; BP/1/6791, partial left postorbital; BP/1/7133, partial left postorbital) demonstrate that the boss is not a peculiarity of individual variation, and that it becomes proportionally larger and more pronounced in larger specimens.

#### Squamosal

No squamosal could be identified among the preserved material.

#### Jugal

Beyond the almost complete left jugal of the holotype, the only pieces of jugal identified among the other material are very short sections bearing the lateral boss (BP/1/6226b, 6232g, h, i, 6734; Fig. 12I, J). In as much as the larger bosses are on deeper (dorsoventrally, not only transversely thicker) pieces of bone, it seems that (absolutely and proportionately) larger bosses were present on larger jugals. In smaller specimens the boss is proportionately smaller and less spherical, but is still considerably more prominent than the low subhorizontal ridge that is present in the same position in *G. prima* ([68]: fig. 3; [23]: plate 11, fig. 6).

#### Quadratojugal

**Figure 13:**
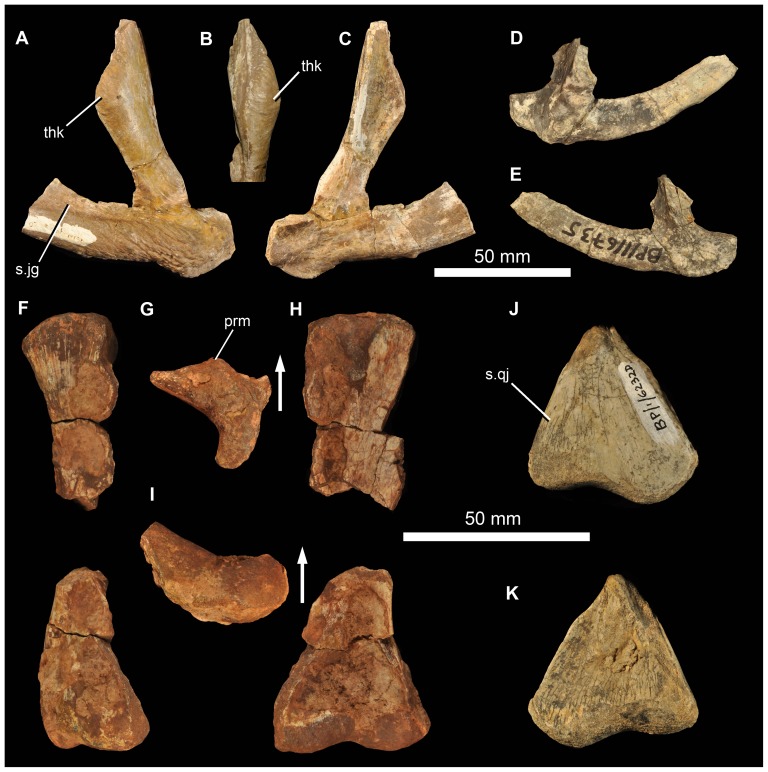
Quadratojugal and quadrate of *Garjainia madiba* sp. nov. A–C, BP/1/7215, left quadratojugal in lateral (A) and anterior (B) and medial (C) views. D–E, BP/1/6735, right quadratojugal in lateral (D) and medial (E) views. F–I, BP/1/5525, incomplete right quadrate (preserved in two pieces) in lateral (F), proximal (G), anterior (H) and distal (I) views. J–K, BP/1/6232d, condyles of right quadrate in posterolateral (J) and anteromedial (K) views. Abbreviations: prm, prominence on anterior surface of proximal quadrate; s.jg, surface for articulation with posterior process of jugal; s.qj, surface for articulation with quadratojugal; thk, thickening on ascending process of quadratojugal. Arrows in (G) and (I) point toward anterior.

The left quadratojugal BP/1/7215 has an acute angle between the ascending and anterior processes seen in lateral view (Fig. 13A–C). The facet for the posterior process of the jugal along the dorsolateral edge of the anterior process is long (Fig. 13A: s.jg). The form of this facet means that the anterior process (which is broken anteriorly) has an approximately teardrop-shaped cross section, being thickened ventrally and tapering dorsally. The lateral surface of the anterior process is notably rugose, especially posteriorly. The quadratojugal is almost excluded from the ventral border of the lateral temporal fenestra, but might have contributed to the very posterior part of this border. The dorsal process of the quadratojugal made a short but notable contribution to the lower part of the posterior border of the lateral temporal fenestra, more similar to the condition in *G. prima* [16], [68] than *E. africanus* [20] or *S. shansisuchus* ([17]: fig. 13; DJG, pers. obs.) in which an elongate ventral process of the squamosal excludes the quadratojugal from the posterior border of the laterotemporal fenestra. The dorsal process of the quadratojugal expands in anteroposterior width towards its dorsal margin. Its posteromedial margin (where it articulated with the quadrate) is broken. Dorsally, the articular surface for the squamosal is marked by a dorsoventrally descending groove on the lateral surface (Fig. 13B). The lateral surface towards the dorsal tip of the ascending process is markedly thickened transversely (Fig. 13A: thk), a feature not documented or seen by us in other erythrosuchids and potentially representing an additional autapomorphy of the new species. A smaller right quadratojugal (BP/1/6735) is generally similar in morphology to BP/1/7215 (Fig. 13D, E).

#### Palate

No unambiguous examples of the vomer, palatine, pterygoid, ectopterygoid, or epipterygoid are represented among the preserved material. Several partial quadrates are preserved (BP/1/5525, partial right quadrate; BP/1/6232e, left quadrate ventral condyle; BP/1/6232d, right quadrate ventral condyle; BP/1/6232f, left quadrate ventral condyle; Fig. 13F–K). The quadrate ventral condyle is somewhat bipartite, with the lateral part drawn out into a flange at its lateral margin (only well-preserved in BP/1/6232d). A short region immediately above the lateral margin of the condyle bears a posterolateral facet that was overlapped by the quadratojugal. The central thickened part of the dorsal end of the right quadrate of BP/1/5525 is also preserved, but is damaged along anterolateral and anteromedial surfaces (Fig. 13F–H). A prominence is present on the anterior surface of the proximal end (visible in dorsal view but extends onto proximal end of anterior edge; Fig. 13G: prm) as in, for example, *E. africanus* (BP/1/4680) and *G. prima* (PIN 951/57). The central rod has a transversely narrow dorsal end and the posteromedial surface is strongly concave dorsally. This best-preserved quadrate is not complete enough to determine its length, nature of possible articulation with the quadratojugal dorsolaterally, or presence/absence of a quadrate foramen.

#### Braincase

**Figure 14:**
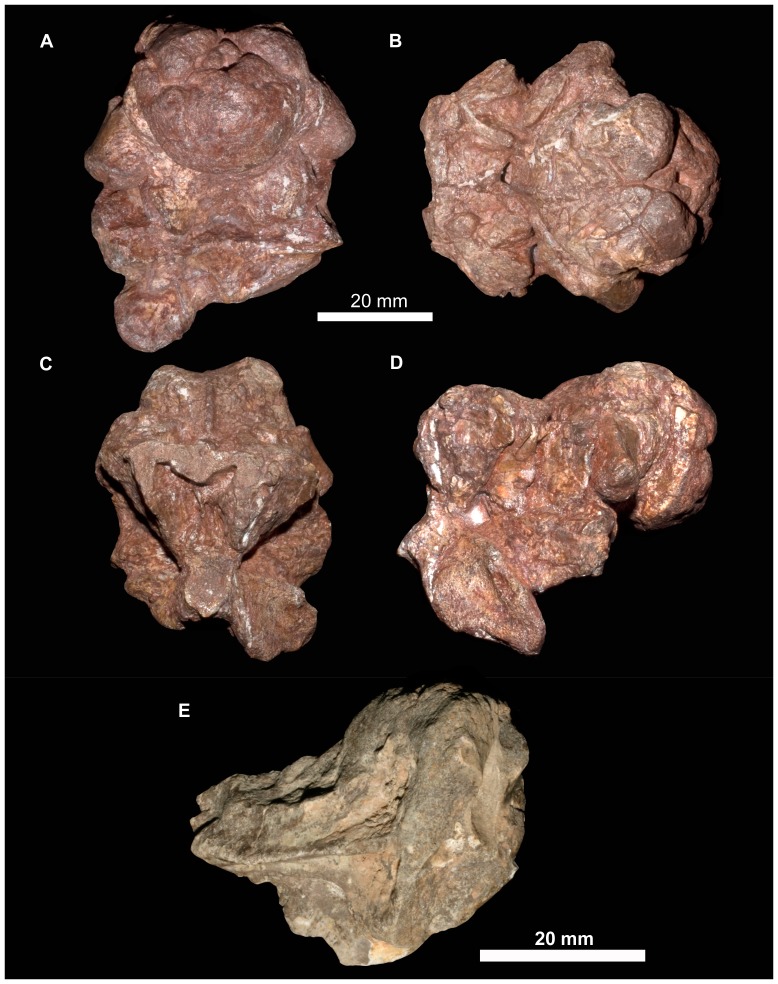
Braincase of *Garjainia madiba* sp. nov. A–D, ventral and posterior part of braincase of BP/1/5525 in posterior (A), dorsal (B), anterior (C) and left lateral (D) views, E, Right posterolateral (and slightly ventral) view of fragmentary parabasisphenoid, BP/1/6232y (the basipterygoid processes and right basal tuber of the parabasisphenoid are missing).

**Figure 15:**
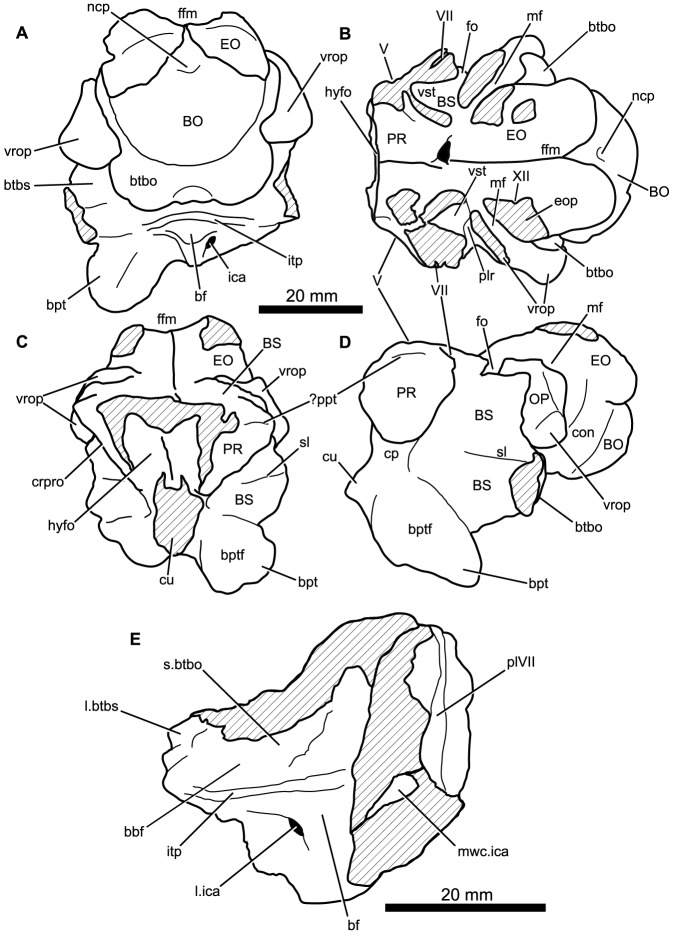
Braincase of *Garjainia madiba* sp. nov. Interpretive line drawings of specimens shown in Fig. 13. A–D, ventral and posterior part of braincase of BP/1/5525 in posterior (A), dorsal (B), anterior (C) and left lateral (D) views, E, Right posterolateral (and slightly ventral) view of fragmentary parabasisphenoid, BP/1/6232y (the basipterygoid processes and right basal tuber of the parabasisphenoid are missing). Abbreviations: bbf, basioccipital-parabasisphenoid fossa; bf, parabasisphenoid fossa; BO, basioccipital; bpt, basipterygoid process; bptf, basipterygoid facet; BS, parabasisphenoid; btbo, basal tuber of basioccipital; btbs, basal tuber of parabasisphenoid; con, condylar neck; cp, clinoid process of parabasisphenoid; crpro, crista prootica; cu, base of cultiform process; EO, exoccipital; eop, exoccipital pillar (separating metotic foramen from fenestra ovalis); ffm, floor of foramen magnum; fo, floor of fenestra ovalis; hyfo, hypophyseal fossa; itp, intertuberal plate; l.btbs, left basal tuber of parabasisphenoid; l.ica, foramen for left cerebral branch of internal carotid artery; mf, floor of metotic foramen; mwc.ica, medial wall of channel transmitting cerebral branch of internal catorid artery; ncp, notochordal pit; plr, pseudolagenar recess; plVII, groove for passage of palatine branch of facial nerve;?ppt, ridge for attachment of *m. protractor pterygoidei*; PR, prootic; s.btbo, surface for articulation with basal tuber of basioccipital; sl, semilunar depression; vrop, ventral ramus of opisthotic; vst, floor of vestibule. V, VII and XII indicate openings for cranial nerves.

There are at least nine fragments of braincases preserved among the new material. Most of these are fragments of parabasisphenoid (e.g., 6232o, v, w, x, y) but there are also multiple fragments of the paroccipital process and parts of the lateral and dorsal walls of the endocranial cavity (BP/1/5760, 6232z, 7342). The most completely preserved piece of braincase is that of BP/1/5525 (Figs. 14, 15). There are no unambiguous laterosphenoids or anterior (cultriform processes of) parabasisphenoids among the preserved material.

**Figure 16:**
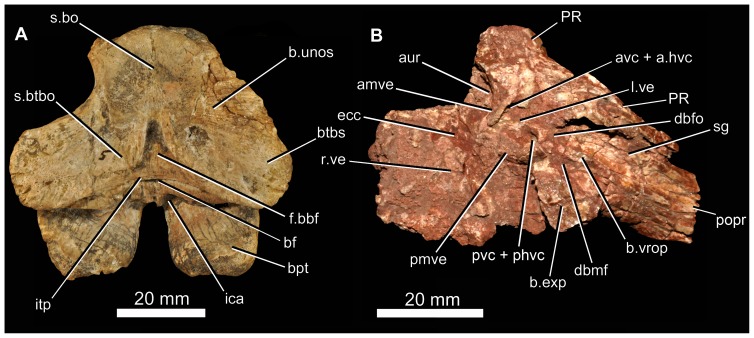
Braincase of *Garjainia madiba* sp. nov. A, posterior view of isolated parabasisphenoid, BP/1/6232w. B, ventral view of centre and left side of central portion of braincase of BP/1/7342 showing proximal end of left paroccipital process and view into ceiling of middle ear region (compare with *G. prima* as figured by Gower & Sennikov ([54]: fig. 3). Abbreviations: a.hvc, anterior opening of horizontal (external) semicircular canal; amve, anteromedial wall of vestibule; aur, auricular recess; avc, opening of anterior semicircular canal; b.exp, break through dorsal end of exoccipital pillar; bf, bparabasisphenoid fossa; bpt, basipterygoid process; btbs, basal tuber of parabasisphenoid; b.unos, base of unossified gap ( =  pseudolagenar recess); b.vrop, break through dorsal end of ventral ramus of opisthotic; dbmf, dorsal border of metotic foramen; dbfo, dorsal border of fenestra ovalis; ecc, ceiling of endocranial cavity; f.bbf, floor of basioccipital-parabasisphenoid fossa; ica, foramen for cerebral branch of internal carotid artery; itp, intertuberal plate; l.ve, ceiling of left vestibule; p.hvc, posterior opening of horizontal (external) semicircular canal; pmve, posteromedial wall of vestibule; popr, paroccipital process; PR, prootic; pvc, opening of posterior vertical semicircular canal; r.ve, ceiling of right vestibule; s.bo, surface for articulation with basioccipital; s.btbo, surface for articulation with basal tuber of basioccipital; sg, stapedial groove.

The braincase (Figs. 7, 14–16) is very similar to that of *G. prima* as described by Gower & Sennikov [54]. The key features include a parabasisphenoid that is verticalized (*sensu* [54]), with the basal tubera lying almost directly above (rather than posterior to) the basipterygoid processes. The lateral surface of the parabasisphenoid above the basipterygoid process bears a semilunar depression (*sensu* [70]). The foramina for the internal carotid arteries are on the posterior surface, and there is a strong intertuberal plate (*sensu* [54]). The basioccipital seemingly lacks the small medial tubercle between the basal tubera seen in *G. prima* [54] but most specimens do not preserve this region and BP/1/5525 is partly eroded here. The ventral ramus of the opisthotic is substantial and clearly visible in posterior and lateral views, and the posterior face of its distal end is tightly and substantially in contact with the lateral edge of the dorsal part of the basal tuber of the basioccipital. The exoccipitals make midline contact on the floor of the endocranial cavity anteriorly, and diverge on the floor of the foramen magnum posteriorly. There appears to be a metotic foramen (i.e., the metotic fissure remains undivided by bone: [71]).

Given the very close resemblance of the braincase of *G. madiba* to that of its Russian congener, the following section summarizes the similarities and differences with respect to *G. prima*.

(1) Similarities. The notochordal pit and condylar “neck” are well defined. The basal tubera of the basioccipital are very similar to those of *G. prima* except perhaps in the absence of a medial tubercle. The large distal end of the ventral ramus of the opisthotic and its relation to the basal tuber of the basioccipital (posteriorly) and an unossified gap (ventrally) are as in *G. prima*. The supraoccipital is probably excluded from the border of the foramen magnum but is clearly exposed further forward on the roof of the endocranial cavity between the thickened anterodorsal parts of the prootics (BP/1/7342). The posterior edges of the clinoid processes of the parabasisphenoid are deeply incised. However, the foramina for the cerebral branches of the internal carotid arteries are on the posterior surface of the parabasisphenoid. Support for the interpretation [54] that the arteries entered the posteroventral surface of the parabasisphenoid rather than behind the clinoid processes (as in many crown-group-archosaurs, [71]) is found in one fragment (BP/1/6232y, Fig. 14E, 15E) in which the channel leading from the putative carotid foramen is seen extending towards the hypophyseal fossa, and lying deeper (further medially) within the bone than the incised posterior border of the clinoid processs. The form of the basal tubera of the parabsisphenoid, the intertuberal plate (e.g., BP/1/5525: Figs. 14, 15; BP/1/6232w: Fig. 16), and the topography of the floor of the endocranial cavity (BPI/1/5525: Figs. 14B, 15B) is as in *G. prima*, and the positions of the abducens and facial nerve channels are also likely very similar except it is unclear precisely how they are positioned relative to the boundary between prootic and parabasisphenoid. The floor (BP/1/5525: Figs. 14B, 15B) and ceiling (BP/1/7342: Fig. 16A) of the vestibule are very similar to those of *G. prima* as far as can be determined.

(2) Differences. Only a single foramen (not two) can be seen for the hypoglossal nerve on each side, although this area is not well preserved in any specimen. The dorsal surface of the supraoccipital is perhaps less prominently and extensively rugose than in *G. prima*. The medial (proximal) end of the posterior surface of the paroccipital process bears a well-defined depression, though this is perhaps in a slightly more medial position than in *G. prima*. The semilunar depression on the lateral surface of the basal tuber of the parabasisphenoid is present, though the central portion is perhaps less incised than in the specimen of *G. prima* figured by Gower & Sennikov [54]. The form of the parabasisphenoid is very similar to that of *G. prima*, but the basisphenoid and basioccipital-basisphenoid fossae either side of the intertuberal plate seem to be relatively smaller (e.g., BP/1/5525, 6232v, 6232w), although there is clearly some intraspecific variation in the details of this region. The anteriorly converging ridges bordering the basisphenoid fossa come to lie immediately behind the foramina for the cerebral branches of the internal carotid arteries, as in *G. prima*; however these ridges mostly converge and meet strongly in the midline, though in two specimens (BP/1/6232o, 6232y) they are lower and more rounded. The vast majority of the cultriform process of the parabasisphenoid is not preserved in any specimen, but in BP/1/5525 its base does not seem to be as tall as the specimen of *G. prima* figured by Gower & Sennikov ([54]: fig. 2C), so that the ventral edge is not as low. There might not be a basioccipital medial tubercle below the occipital condyle and between the basal tubera of the basioccipital, though this region is not well preserved in BP/1/5525 (Fig. 14A).

(3) Extent of similarity or difference unclear. The exoccipitals diverge posteriorly along the top of the basioccipital (floor of the foramen magnum) in *G. prima* [54] and perhaps slightly less strongly in *G. madiba* although the small amount of disarticuation in this region of BP/1/5525 makes this a little unclear. The nature of the contact between supraoccipital and parietal/postparietal is unclear in *G. madiba*, and it is unclear whether or not there is a narrow subhorizontal ridge on the lateral surface of the prootic below the trigeminal foramen or whether the edge of crista prootica is curved simply (right side of BP/1/5525) or is slightly sinusoidal (perhaps left side of BP/1/5525) in lateral view. The number of external foramina for the facial nerve is unclear in *G. madiba*, and the laterosphenoid is not known. The floor of the endocranial cavity is not well preserved in BP/1/5525, so detailed comparisons with *G. prima* cannot be made, including the presence of nutrient foramina, the exact form of contacts between elements, and the position and form of perilymphatic foramen, but the general configuration in *G. madiba* is clearly similar to that of *G. prima*. Similarly imperfect preservation of the roof and walls of the endocranial cavity (including the degree of ossification of the medial walls of the vestibule) in the *G madiba* material limits detailed comparison between the species, though there are no obvious differences to the condition in *G. prima*. Contrary to a previous description ([54]: 886) we strongly doubt that the laterosphenoid of *G. prima* was likely excluded from the border of the trigeminal foramen. The condition in *G. madiba* is not clear based on the available material.

#### Dentary

**Figure 17:**
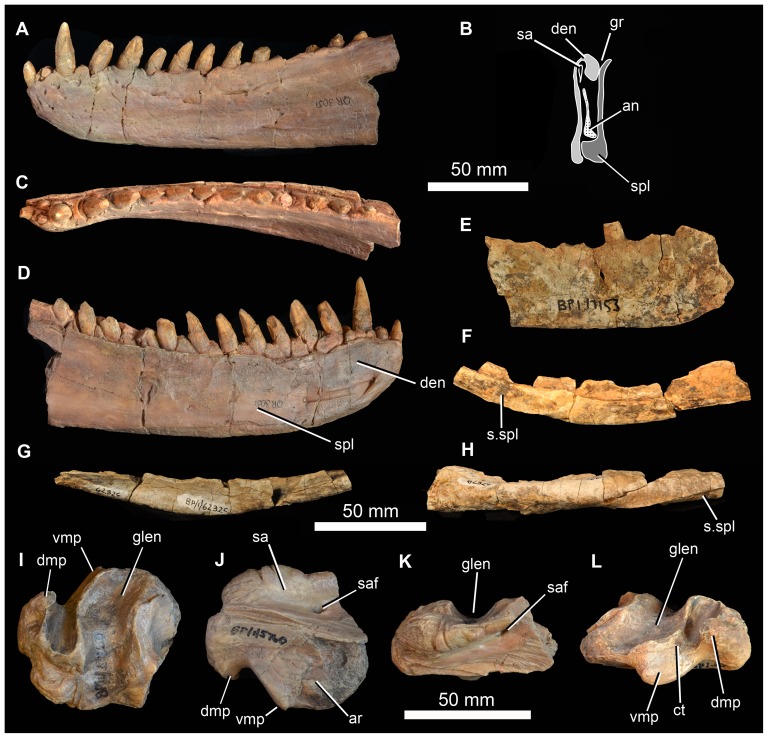
Mandible of *Garjainia madiba* sp. nov. A–D, cast of anterior part of left mandible of NMQR 3051 in lateral (A), posterior (B), dorsal (C) and medial (D) views. E, lateral view of anterior part of right dentary, BP/1/7153; F–H, right angular, BP/1/6232c in medial (F), lateral (G) and ventral (H) views. I–L, cast of right articular and posterior part of surangular, BP/1/5763b (*contra* handwritten label on cast) in dorsal and slightly lateral (I), ventral and slightly medial (J), lateral and slightly ventral (K) and medial and slightly posterodorsal (L) views. Abbreviations: an, angular; ar, articular; den, dentary; dmp, dorsomedial process of retroarticular region; glen, glenoid; gr, groove possibly for articulation with coronoid; sa, surangular; saf, surangular foramen; spl, splenial; s.spl, surface for articulation with splenial; vmp, ventromedial process of articular.

The best-preserved dentary (NMQR 3051), a left example, is laterally concave along its longitudinal axis and has 14 alveoli, mostly holding complete or partially preserved teeth (Fig. 17A–D). There is no clear evidence of additional alveoli having been present behind the 14th. The apicobasally tallest preserved tooth lies in position 3 (followed closely by 4 and 5). The first three teeth are slightly procumbent, whereas more posterior crowns project posterodorsally. Anteriorly the symphyseal region is not notably differentiated from the remainder of the medial surface of the bone. In lateral view, the anterior end of the dentary is not dorsoventrally expanded in outline, but instead tapers slightly towards its anterior termination. The external surface bears a number of nutrient foramina, the largest of which form a series of three openings that lie along the anterior edge of the dentary, ventral to the first three teeth and slightly below the alveolar margin (exactly the same in NMQR 3049); there are foramina here also in *G. prima* but with variation in the number, size and exact position. Medially there is a distinct step a short distance ventral to the alveolar margin. Dorsal to this step are a series of well-preserved, triangular interdental plates, one present between each pair of adjacent teeth. Two additional partial dentaries are present in the Johannesburg collection (BP/1/6232k and BP/1/7153, Fig. 17E), and an additional anterior fragment of a right dentary in the NM collection (NMQR 3049). It is clear from the medial surface of the dentary of BP/1/7153 that the Meckelian canal was held primarily by the dentary and that it tapered in depth (transversely) and height posterior to the symphyseal region. The most complete dentary known for *G. madiba* (NMQR 3049) is notably more curved in dorsal and ventral view (laterally concave, medially convex) than examples of this element of *G. prima*. Taken at face value, this would indicate that the mandibles of *G. madiba* were relatively shorter and at a greater angle to one another than in *G. prima*, but the alternative explanation of differential deformation during preservation cannot be ruled out.

#### Splenial

The only unambiguous splenial is preserved in articulation with the left dentary as part of NMQR 3051 (Fig. 17B, D). Posteriorly it is almost as deep as the dentary, extending from the ventral margin of the mandible to the alveolar margin. For most of its length, however, the splenial does not extend to the ventral mandibular margin. Posteriorly the splenial is gently concave along its vertical axis, but this might at least partially represent collapse into the Meckelian canal. The posterior end of the splenial has an L-shaped cross section ventrally, while the dorsal edge curves away dorsomedially to form a V-shaped groove between it and the dentary (Fig. 17B). This groove possibly held the coronoid, if this was elongate as in *G. prima* [68] and *E. africanus* [20]. The mid section and anterior end of the splenial maintain a dorsomedially curved dorsal edge, but the ventral edge is more planar (not L-shaped). The anterior end of the splenial is incompletely preserved, but it seems likely that it terminated posterior to the symphysis.

#### Coronoid and prearticular

No unambiguous examples are known. A small fragment of prearticular is probably preserved in contact with the left articular of NMQR 3049, but nothing of note is worth describing. A dorsally open groove between the dentary and splenial (Fig. 17B) likely held an elongate coronoid, as in *G. prima* ([68]: fig. 6).

#### Surangular and articular

The only unambiguous partial surangulars are posterior fragments preserved tightly in articulation with the corresponding articulars (NMQR 3049 left and right; BP/1/5763b (Fig. 17I–L) right; 6232b left; 7340 right; 7153 left; JNN 99.4.30 left). This region of the mandible does not seem to differ notably from that of other erythrosuchids. The retroarticular process of the articular comprises a transverse concavity (perhaps more concave than in *G. prima*) immediately behind the cotyle, and behind this a short, thickened process that bears a laterally compressed, hook-like ascending process ( =  “dorsomedial projection” of Nesbitt [7]; Fig. 17I–J, K: dmp) on its posteromedial corner. This process ascends more prominently and is more hooked than in *E. africanus* [20], but is very similar to that of *G. prima* ([68]: fig. 6). In all specimens where this region is preserved and prepared there is a small foramen immediately beyond the anteromedial corner of the transverse concavity (Fig. 17K: ct), this might represent the route of the chorda tympani branch of the facial nerve. As in *G. prima* and *E. africanus* there is a short, robust anteroventromedially projecting tubercle medial to the posterior corner of the cotyle (Fig. 17I–J: vmp;  =  “ventromedial process” of [7]). As in other erythrosuchids, this is not dorsoventrally compressed and dorsally does not carry a medial extension of the concavity between the cotyle and the retroarticular process such as is seen in, for example, at least some rauisuchians (e.g. [72]). The anterior end of the medial face of the articular bears a facet for the prearticular. Level with the posterior end of this facet, the surangular bears a large surangular foramen (Fig. 17J, K: saf).

#### Angular

There are at least two elongate, shallowly angled elements that likely represent incomplete angulars of *G. madiba* (BP/1/5525, left? and BP/1/6232c, right: Fig. 17F, G). The medial surface is less tall than the lateral surface. Posteriorly, the element is transversely flattened, where it would overlap the surangular, and anteriorly the medial surface bears a shallow, posteriorly tapering concavity that perhaps represents a facet, probably for the splenial (Fig. 17G: s.spl). The posterior surface of the anterior end of the left mandible of NMQR 3051 shows the probable anterior end of the angular, slightly L-shaped, with a small ventromedial projection that lies above the ventrolateral projection of the splenial, and a dorsally tapering lateral blade lying close to the medial surface of the dentary (Fig. 17B). The central region of the mandible is not well known in proterosuchians (*sensu* [10]) and further interpretation of fragmentary and disarticulated material is not currently possible with confidence.

#### Dentition

The dentition is clearly thecodont with deep-rooted teeth held in well-defined sockets (Figs. 9, 10, 11, 17). There is some variation in the tightness of contact between tooth and bone among specimens, but also along the tooth-bearing margin of a single element and even around the perimeter of a single tooth. For example, although the less fully erupted maxillary teeth of BP/1/5525 are thecodont, the more fully erupted teeth are ankylothecodont. In our view the instances of close bone-tooth association are not prevalent or strongly developed enough for the implantation as a whole to be considered ankylothecodont, and the dentition is overall more thecodont than in, for example, *Proterosuchus* and *Sarmatosuchus* [69]. The long tooth roots are subcircular to oval in cross section. The crowns are only moderately laterally compressed and recurved (e.g. BP/1/7153). Both anterior and posterior edge of teeth are serrated. There are four to five denticles within 1 mm on a replacement maxillary tooth in BP/1/5525 (Fig. 11I) and a similar number in a premaxillary tooth of BP/1/6232n (Fig. 9G). Preserved replacement teeth emerge on the medial side of functional teeth.

#### Intercentra

**Figure 18:**
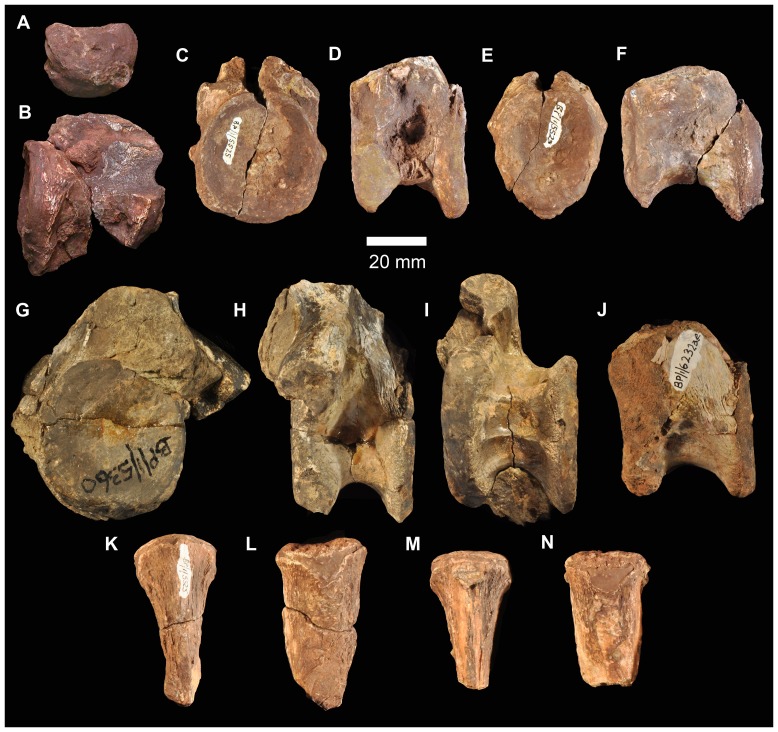
Cervical vertebrae of *Garjainia madiba* sp. nov. A, atlantal intercentrum of BP/1/5525 in anterior view. B, axial centrum of BP/1/5525 in left lateral view. C–F, postaxial cervical centra of anterior cervical vertebrae of BP/1/5525 in anterior (C, E) and left lateral (D, F) views. G–I, posterior cervical centrum of BP/1/7338, anterior cervical vertebra in anterior (G), left lateral (H), and ventral (I) views. J, BP/1/6232ae, anterior cervical vertebra in left lateral view. K–N, BP/1/5525, cervical neural spines in anterior or posterior (K, M) and lateral (L, N) views.

**Figure 19:**
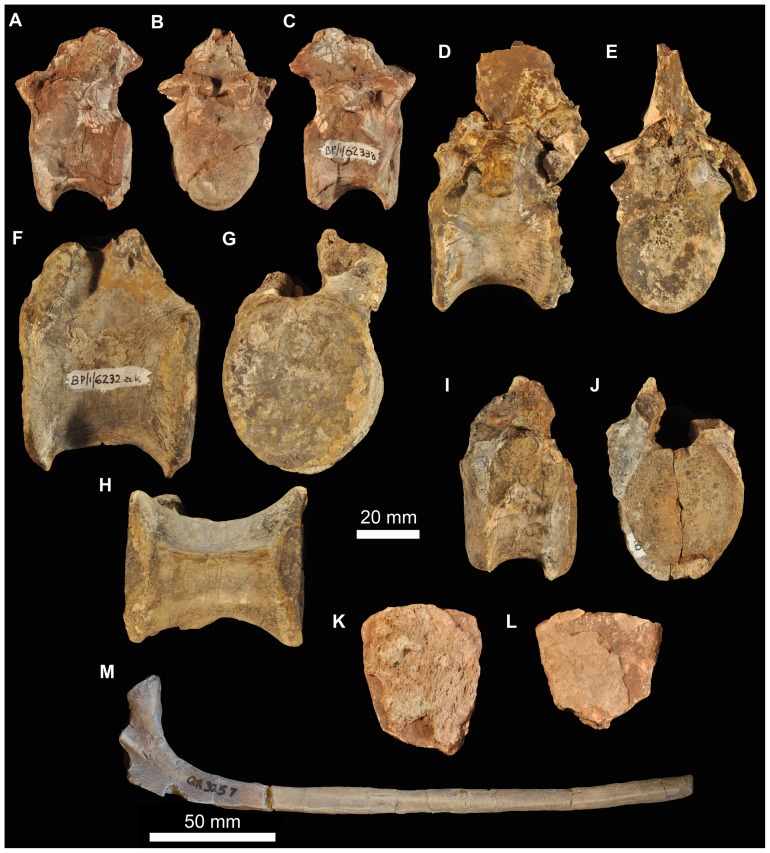
Dorsal vertebrae and ribs of *Garjainia madiba* sp. nov. A–C, BP/1/6233b, anterior to mid-dorsal vertebra in left lateral (A), anterior (B) and right lateral (C) views. D–E, BP/1/7135, anterior to mid-dorsal vertebra in left lateral (D) and anterior (E) views. F–H, BP/1/6232ak, mid to posterior dorsal vertebra in left lateral (F), anterior (G), and ventral (H) views. I–J, BP/1/6232aj, posterior dorsal vertebra in left lateral (I) and anterior (J) views. K–L, BP/1/5525, neural spine fragments. M, cast of three-headed rib, NMQR 3257.

Other than the atlas-axis complex there are no intercentra identified among the preserved material. Intercentra were likely present throughout the presacral column, based on ventrally bevelled centra (Figs. 18, 19) and comparison with other proterosuchians (e.g., *Proterosuchus fergusi*: [73]; *E. africanus*: [20]).

#### Cervical vertebrae

Part of the atlas-axis complex is preserved in BP/1/5525 (Fig. 18A–B). The atlas intercentrum is approximately semicircular (in anterior and posterior views), with a bevelled posterior ventral edge and a single lateral facet for a rib on each side. The axis is broken and preserves only part of the centrum, the anterior end of which is typical for early archosauriforms in having a vertical articular face dorsally, probably for the atlas centrum, and a large bevelled region below this for articulation with an intercentrum. There are no rib apophyses low on the anterolateral edge of the axis centrum, and the likely area for the diapophysis (dorsally on the lateral edge of the anterior end of the centrum) and facet for articulation with the atlas neural arch is not very well preserved or prepared.

The only vertebra found close to the holotype material is a broken anterior cervical (BP/1/5760b), with the neural spine mostly missing and the remaining part of the neural arch largely detached from the centrum. It is one of the anteriormost cervical vertebrae, as indicated by the position of the parapophysis at the ventral end of the anterior edge of the centrum with the diapophysis a short distance above this. The centrum is marginally longer than it is tall, with the articular faces somewhat offset from each other, the top and bottom of the posterior face lying a little lower than those of the anterior face. The ventral edge is moderately arched in lateral view and the body of the centrum is waisted in ventral view. The base of the neural spine is much shorter than the length of the centrum and lies towards the posterior end of the neural arch.

Multiple other cervical vertebrae are present (BP/1/5525, BP/1/6232ae, ai, al, BP/1/7234, 7327), but in nearly all cases only the centrum and the base of the neural arch are preserved (Fig. 18). Two anterior cervical centra preserved as part of BP/1/5525 (Fig. 18C–F) are strongly waisted/pinched and approximately as long as they are tall (also in BP/1/7327). Their apophyses are positioned far anteriorly, the parapophyses far ventrally and the diapophyses a short distance below the dorsal edges of the centra. The more anterior of these two centra (with a more ventrally positioned diapophysis) has a single midventral ridge extending back to a little beyond halfway from the anterior edge of the centrum, fading out a little in front of a small triangular (tapering anteriorly) rugosity that is continuous with the ventral margin of the posterior articulatory surface of the centrum. The more posterior example has a relatively smooth, flat and featureless ventral surface. The faces of the cervical centra are subcircular, perhaps taller than wide in the more anterior example, and they are offset with the posterior face extending further ventrally than the anterior one. An anterior cervical in BP/1/5360 has low ventral midline keel/ridge.

More posteriorly within the cervical column the centra are more strongly waisted and relatively shorter (and taller) and the two articulatory faces are slightly less offset. In addition the diapophysis is positioned further dorsally and posteriorly, away from the centrum and on a short ventrolateral process of the neural arch. There are no obviously posterior cervical neural arches closely resembling the ‘pectorals’ (*sensu* [20]) preserved among the vertebral remains. However, a couple of disarticulated centra with partial neural arches, BP/1/7338 (Fig. 18G–I) and BP/1/6232al, are perhaps from this region because they are short (anteroposteriorly), deeply waisted, have diapophyses on laterally projecting processes and parapophyses still low down and just behind the anterior face of the centrum. There is no evidence for three-headed ribs in the ‘pectoral’ region based on the fragmentary vertebral remains, though the presence of this feature is indicated by an incomplete rib (see below).

Several relatively short (anteroposteriorly), broken neural spines preserved as part of BP/1/5525 are possibly from cervical vertebrae because they are transversely expanded distally. The distal ends vary in outline from circular to oval, and they have rugose surfaces that extend onto the distal end of the lateral surfaces of the spines.

#### Dorsal vertebrae

The preserved dorsal centra are all constricted, and bevelled ventrally for intercentra. BP/1/7135 (Fig. 19D–E) and 6233b (Fig. 19A–C) are anterior to middorsals, incompletely prepared and lacking parts of the zygapophyses as well as the neural spines. The centra are marginally longer than tall with oval faces. The parapophysis is anteriorly positioned at the top of the face of the centrum, the diapophysis is behind and above this on the neural arch with a very low paradiapophyseal lamina between the two apophyses (the dorsal proximal part of the two headed rib is in articulation on the left of BP/1/7135). Anterodorsal to the apophyses are low prezygadia- and prezygaparapophyseal laminae that bound a shallow prezygapophyseal centrodiapophyseal fossa. There are also spinodiapophyseal and centrodiapophyseal fossae. There are no (or very minor) laminae posterior to the diapophysis. The position of incomplete dorsal vertebrae was estimated by comparison with other erythrosuchids, especially *E. africanus* and *G. prima*.

BP/1/6232ak is a mid to posterior dorsal. The centrum is about as long as tall with oval to subcircular faces. The parapophysis is still distinct from the diapophysis but more dorsally positioned than in the two dorsals described immediately above. The diapophysis and most of the neural arch is not preserved, but it appears to have strongly overhung the centrodiapophyseal fossa.

BP/1/6232aj is possibly a posterior dorsal with a centrum that is as long as tall. It has a confluent para- and diapophysis without prezygadia- or prezygaparapophyseal laminae or a prezgapophyseal centrodiapophyseal fossa. There is no notable posterior centrodiapophyseal lamina, and a low postzygadiapophyseal lamina and a spinodiapophyseal fossa. This vertebra resembles that of *E. africanus* figured by Gower ([20]: fig. 24E).

#### Sacral vertebrae

**Figure 20:**
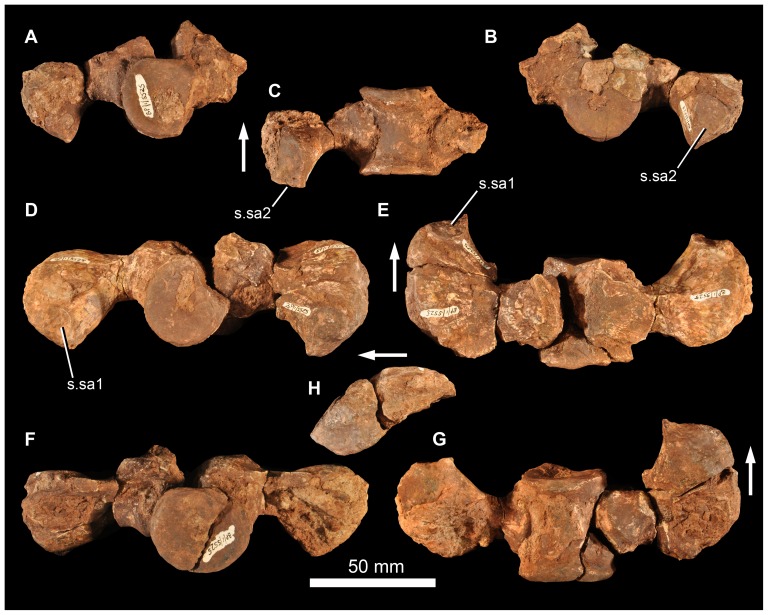
Sacral vertebrae and ribs of *Garjainia madiba* sp. nov., BP/1/5525. A–C, sacral vertebra 1 in anterior (A), posterior (B) and ventral (C) views. D–H, sacral vertebra 2 in anterior (D), dorsal (E), posterior (F), and ventral (G) views, and distal end of left sacral rib (H). Arrows in (C), (E), (G) and (H) point toward anterior. Abbreviations: s.sa1, surface for articulation with sacral rib 1; s.sa2, surface for articulation with sacral rib 2.

Both of the two sacral vertebrae of BP/1/5525 are preserved (Fig. 20), and are not fused to one another. The first sacral vertebra is smaller than the second (with a shorter and less broad centrum: 33 mm long), and only its right rib is well preserved. The centrum is spool-like, with no ventral ridge or groove, and with shallowly concave, subcircular articular faces. The large rib attaches to the central part of the vertebra, and extends laterally and slightly ventrally. The distal end of the rib is broadly expanded anteroposteriorly and the distal surface is convex in dorsal view, and formed a broad attachment with the ilium slightly posterior to the point of contact between the pubic peduncle and preacetabular process of the dorsal blade. At the posterior margin of its distal end the rib has an articular surface that contacted the rib of the second sacral vertebra. The second sacral centrum is larger than the first (42 mm long), is also spool-shaped, and has a very large rib attaching to the central part of the vertebra across the neurocentral junction. This rib also extends laterally and slightly ventrally and at its distal end is massively expanded into an articular surface that in lateral view is arched posteroventrally. This rib articulated with the ilium medial to the base of the postacetabular process of the dorsal blade and the posterior part of the acetabulum, and also articulated with the rib of the first sacral vertebra. The second rib has a groove on its distal articular surface that matches a ridge within the sacral rib facet of the ilium.

An incomplete larger sacral vertebra and rib is preserved in BP/1/6738. This is interpreted as a first sacral. The base of the rib extends to the anterior edge of the centrum and forms an additional articular surface alongside the anterior central face, very much as in the large *E. africanus* specimen BMNH R3592 and unlike the smaller *G. madiba* material of BP/1/5525, suggesting that this feature might vary with ontogeny.

#### Caudal vertebrae

**Figure 21:**
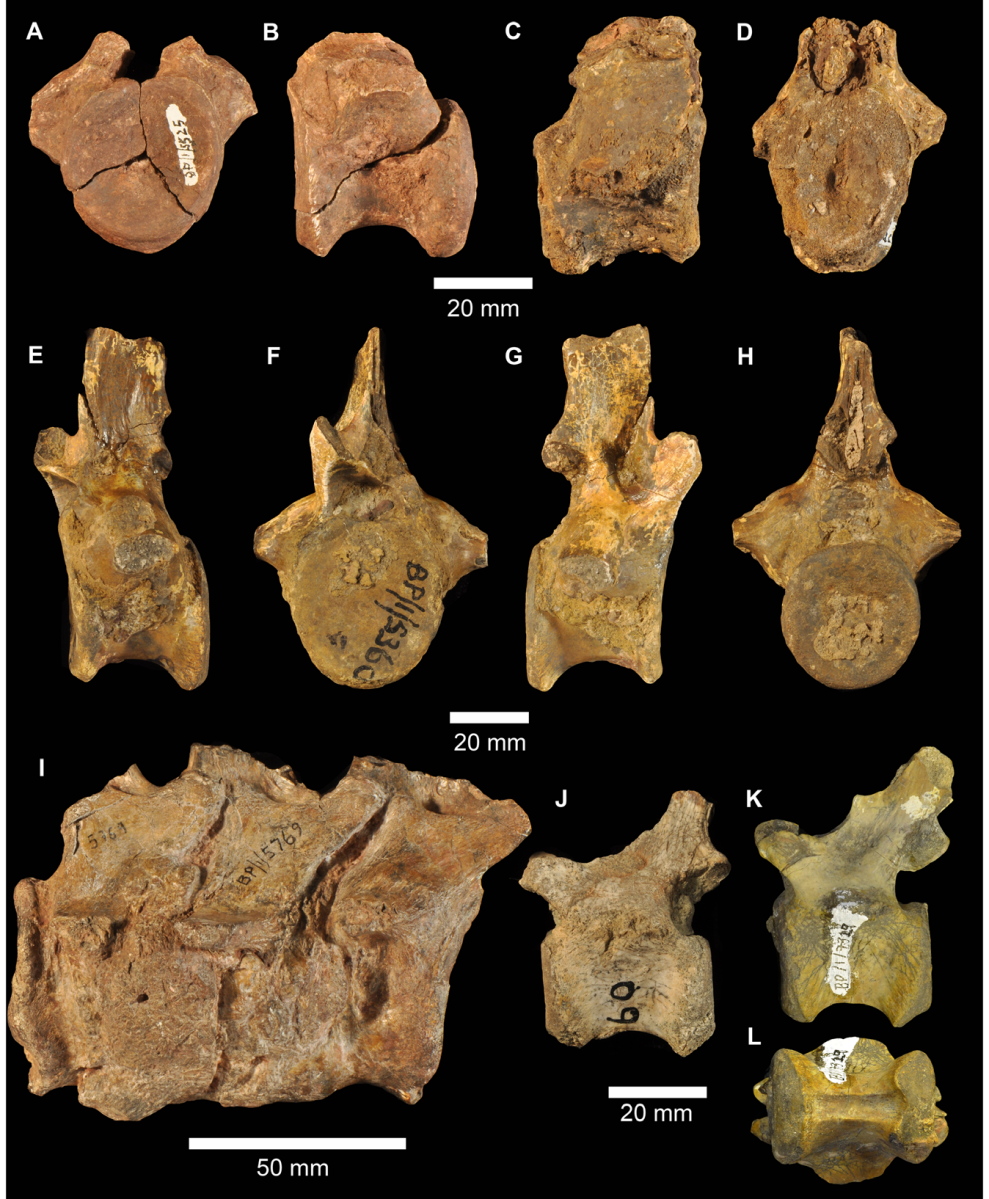
Caudal vertebrae of *Garjainia madiba* sp. nov. A–B, BP/1/5525, anterior caudal vertebra in anterior (A) and left lateral (B) views. C–D, BP/1/6232ac, anterior caudal vertebra in right lateral (C) and anterior (D) views. E–H, BP/1/7335, anterior caudal vertebra in left lateral (E), anterior (F), right lateral (G), and posterior (H) views. I, BP/1/5769, three fused anterior caudal vertebrae in right lateral view. J, BP/1/7334, mid to posterior caudal vertebra in left lateral view (J). K, L, BP/1/7329, mid to posterior caudal vertebra in left lateral (K) and ventral (L) views.

BP/1/5525 includes an anterior caudal centrum (Fig. 21A–B), and BP/1/6232ac (Fig. 21C–D) is a more complete but less well preserved anterior caudal. The best preserved anterior caudal is BP/1/7335 (Fig. 21E–H), which bears a close resemblance to the approximately homologous element of *E. africanus* figured by Gower ([20]: fig. 26). Its centrum is not strongly constricted, marginally taller than long, with a posterior face that extends ventrally beyond the level of the anterior face; both faces are subcircular and with weak to moderate ventral bevels. The proximal end of a single-headed, stout caudal rib is firmly attached centrally in the region where the centrum-arch suture probably lies. There are no notable laminae surrounding the rib apophysis, and the only notable lateral fossa is spinodiapophyseal. Posteriorly there is a well-developed spinopostzygapophyseal fossa.

**Figure 22:**
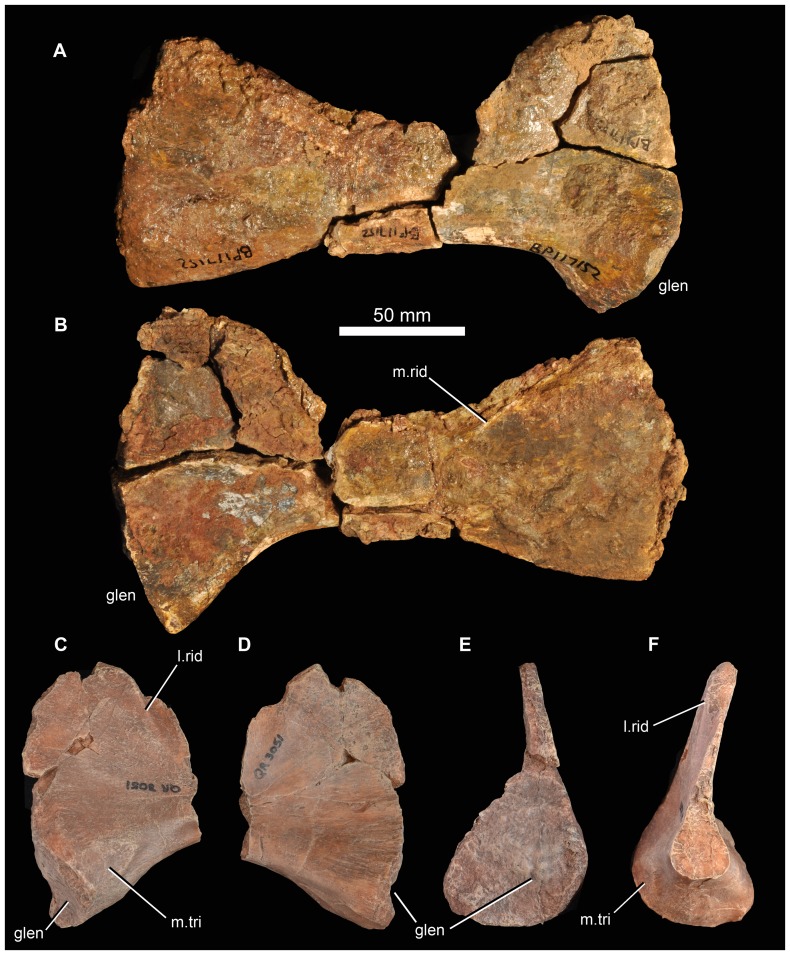
Scapula of *Garjainia madiba* sp. nov. A–B, BP/1/7152, right scapula in lateral (A) and medial (B) views. C–F, cast of proximal end of left scapula of NMQR 3051 in medial (C), lateral (D), ventral (E) and dorsal (F) views. Anterior is to the top of the page in all cases. Abbreviations: glen, glenoid; l.rid, lateral ridge above acromion; m.rid, ridge on medial surface of blade; m.tri, scar of probable origin of *m. triceps*.

**Figure 23:**
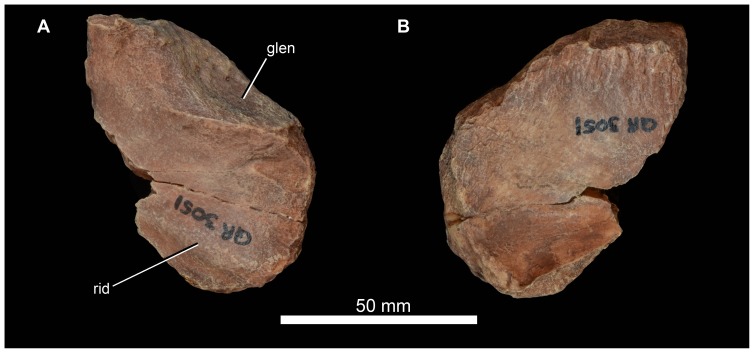
Coracoid of *Garjainia madiba* sp. nov. Cast of partial left coracoid of NMQR 3051 in lateral (A) and medial (B) views. Abbreviations: glen, glenoid; rid, ridge.

**Figure 24:**
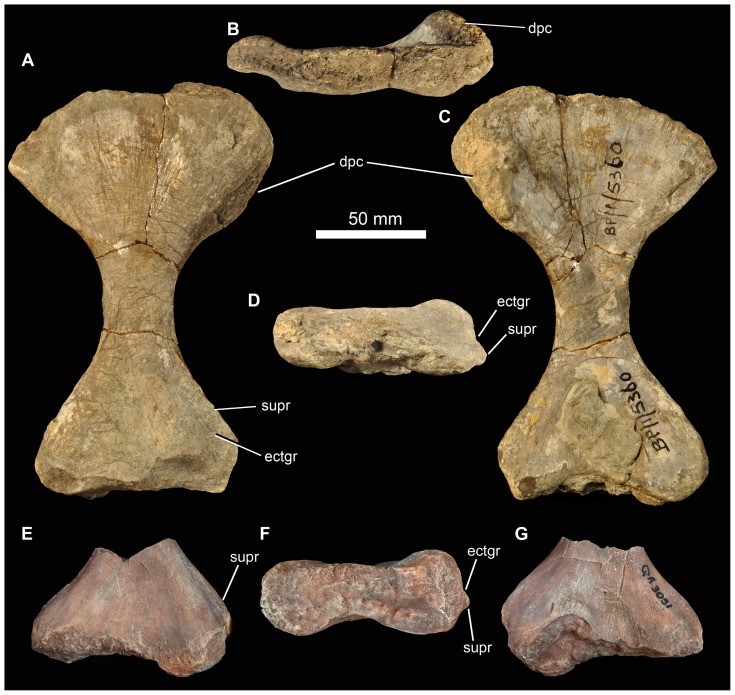
Humerus of *Garjainia madiba* sp. nov. A–D, BP/1/7336, right humerus in dorsal (A), proximal (B), ventral (C) and distal (D) views. E–G, cast of distal end of right humerus of NMQR 3051 in dorsal (E), distal (F) and ventral (G) views. Abbreviations: dpc, deltopectoral crest; ect, ectepicondylar groove; supr, supinator ridge.

**Figure 25:**
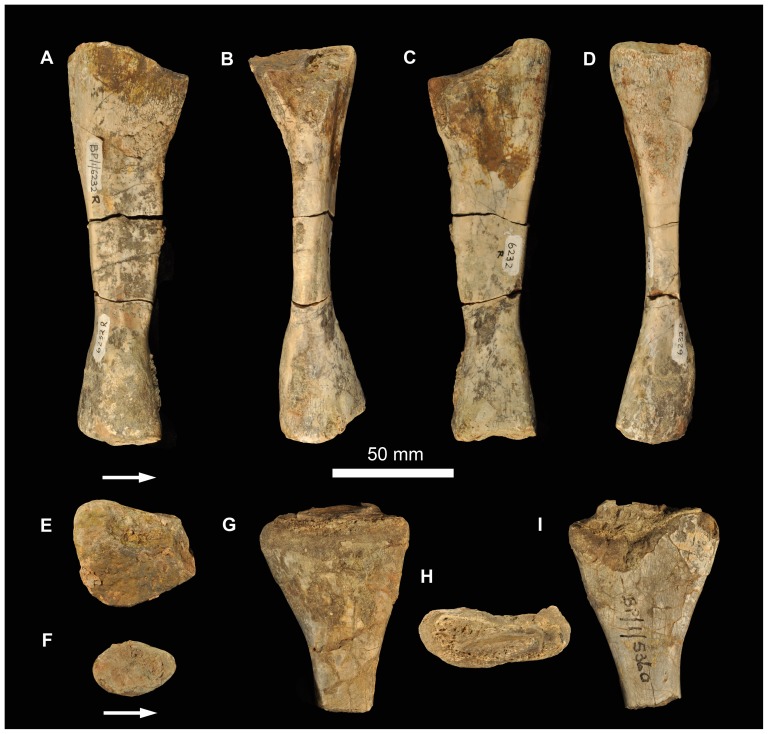
Ulna, radius of *Garjainia madiba* sp. nov. A–F, BP/1/6232r, right ulna in lateral (A), dorsal (B), medial (C), and ventral (D), proximal (E) and distal (F) views. G–I, BP/1/7325, possible proximal end of left radius in ventral (G), proximal (H) and dorsal (I) views. Arrows point to dorsal/anterior in E and F.

**Figure 26:**
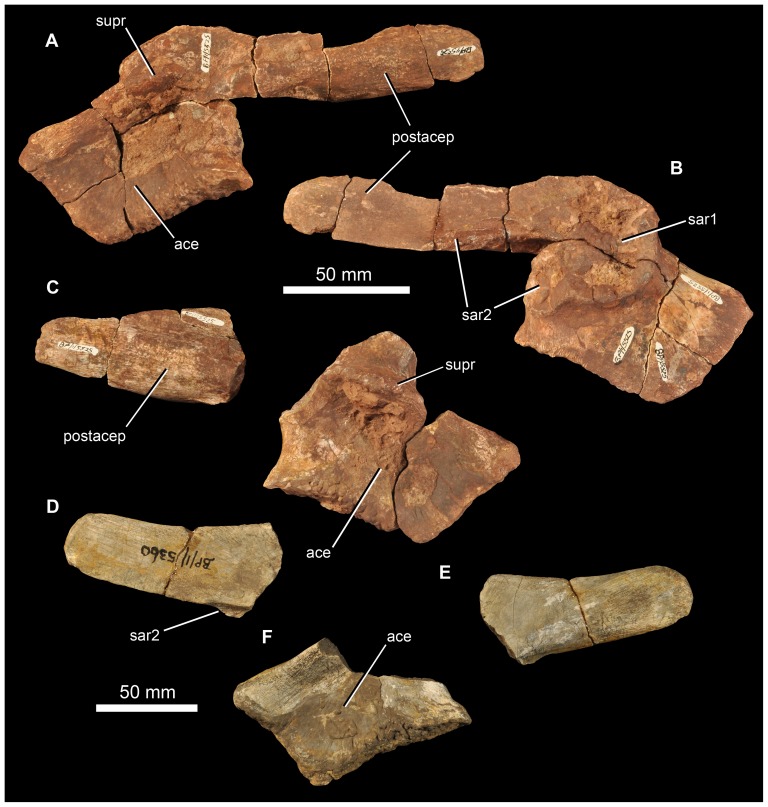
Ilium of *Garjainia madiba* sp. nov. A–B, BP/1/5525, left ilium in lateral (A) and medial (B) views. C, BP/1/5525, right ilium in lateral view. D–F, BP/1/7333, left ilium, postacetabular region in medial (D) and lateral (E) views, and acetabular region in lateral view (F). Abbreviations: ace, acetabulum; postacep, postacetabular process; sar1, sar2, facets for articulation with sacral ribs 1 and 2; supr, supracetabular rim.

BP/1/5769 comprises three large, incomplete anterior caudals with the centra fused together pathologically (Fig. 21I). The neural spines are broken but the rest of the arches are preserved, as are the bases of the ribs, the anteriormost one of which is slightly posterolaterally directed (the other two more lateral) and all three somewhat dorsoventrally compressed. The proximal end of a haemal arch is preserved at least between the second and third of these vertebrae.

Other caudal vertebrae are not well represented and there is no indication of how long the complete tail might have been. BP/1/7329 is the best preserved likely mid to posterior caudal (Fig. 21K, L). The centrum is substantially longer than tall and only moderately constricted, with strongly bevelled ventral edges to the articulatory faces. The posterior bevel is B-shaped for articulation with a haemal arch and there is a low double keel. The base of a small, single-headed rib lies centrally, slightly above the top of the body of the centrum. The neural spine projects posterodorsally and the anterior margin of its base bears a small accessory spine, and there is a shallow spinodiapophyseal fossa. BP/1/7334 is a slightly less completely preserved but very similar vertebra (Fig. 21J) in which the accessory neural spine is broken or absent, and the spinodiapophyseal fossa slightly better developed.

There are some isolated caudal vertebrae that might more tentatively be referred to *G. madiba* on the basis of superficial similarity to *E. africanus* and the lack of evidence of more than one erythrosuchid from this geological unit, but these are incomplete and disarticulated and do not provide much information other than having longer, lower centra (approaching twice as long as tall) with strongly bevelled edges and low double keels, and having smaller but still centrally positioned rib bases.

#### Ribs

A probable rib shaft fragment is preserved among the holotype material (Fig. 8), but the best-preserved presacral rib (NMQR 3257, Fig. 19M) provides evidence suggestive of a tripartite contact with its corresponding vertebra, but no vertebrae with matching apophyses are currently known. Three-headed ribs are known in some Triassic archosauriforms, including the erythrosuchids *G. prima* and *E. africanus* [20].

#### Pectoral girdle

A relatively complete, but fragmented and poorly preserved, right scapula is known (BP/1/7152: Fig. 22A–B). The far distal end is incomplete. The anterior margin is broken and poorly preserved, and the acromion region is very poorly preserved. The long axis of the element is slightly bowed, the medial surface concave, the lateral convex. Proximally the scapula is tapered anteriorly and strongly thickened posteriorly, with a concave ventrally facing glenoid. The posterior margin of the blade (above the expanded proximal end) is nearly straight in lateral view. The precise narrowness of the neck of the blade and the degree of expansion of the distal end cannot be determined accurately because of damage to the anterior margin. There is a low ridge on the medial surface of the blade, close and subparallel to the preserved anterior margin and slightly distal to the midpoint of the preserved length, similar to the condition in *E. africanus* ([20]: fig. 29A) and *G. prima* (PIN 2394/5 and 951/4). The proximal end of a left scapula of NMQR 3051 (Fig. 22C–F) is better preserved, and the posteroventral corner of the lateral surface has a roughened surface, likely for the origin of the *m. triceps* (Fig. 22C: m.tri). The acromion region of NMQR 3051 shows a marked transition along the anterior edge from a thicker more proximal part to thinner more distal part, there being a subhorizontal ridge here that extends back from the anterior edge of the lateral face (Fig. 22C: l.rid). The scapula of *G. madiba* is very similar to that of *G. prima* in all respects. The overall form (concave anterior margin in lateral view, expanded distal end) of the scapula of *Garjainia* is like that of *Shansisuchus* [17], [65] and *E. africanus* [15], [20] and unlike that of *Proterosuchus* [73] and *Sarmatosuchus otschevi* [69].

NMQR 3051 includes a partial left coracoid (Fig. 23) that matches in size the partial scapula of the same specimen. The element as a whole was likely approximately semicircular, generally concave medially and convex laterally, transversely fairly thick dorsally, and preserves the broad and largely featureless likely ventral part of the glenoid (Fig. 23A: glen). Immediately below the posterior end of the glenoid, the lateral surface of the coracoid bears a small scar-like feature, and further below this there is a lateral ridge along a somewhat thickened region (Fig. 23A: rid). In all respects the coracoid is very similar to that of *G. prima* ([9]; PIN 2394/5). The clavicles and interclavicle of *G. madiba* are unknown among the available material.

#### Humerus

The humerus is known from three right-sided examples: an almost complete element (with damage to the proximal and distal ends, BP/1/7336: Fig. 24A–D), a similarly sized distal end (NMQR 3051: Fig. 24E–G) and larger midshaft (BP/1/6233a). These closely resemble each other and the humeri of *E. africanus* [20], *Shansisuchus* [17], [65] and *G. prima* ([9]; PIN 951/36). The deltopectoral crest of *G. madiba*, even in the largest specimen, is proportionally much smaller than in the large holotype and BMNH R3592 of *E. africanus* ([74]: figs. 1, 2; [75]: fig. 13; [20]: fig. 30) in that it is less continuous with proximal terminal end, less prominent, and extends shorter distance down shaft. The prominent part of the deltopectoral crest of *G. madiba* has a rugose edge and extends about one third down the length of the humerus in BP/1/7336.

The long axes of the proximal and distal ends of the humerus of *G. madiba* are twisted relative to one another at approximately 30 degrees. The midshaft is dorsoventrally compressed relative to its width (depth is two thirds of width). Distally there is a well-developed lateral supinator ridge (Fig. 24: supr) that extends away proximally from the supinator process bordering the ectepicondylar groove (Fig. 24: ectgr). The lateral part (ectepicondyle) of the distal end is more expanded than the medial part (entepicondyle).

#### Ulna and radius

A large right ulna (BP/1/6232r, Fig. 25A–F) is similar to that of *G. prima, S. shansisuchus* and *E. africanus*, though slightly more slender than the larger holotype of the latter ([74]: fig. 7; [20]: fig. 31). The proximal end is subtriangular and notably larger than the oval distal end. The anterior edge (*sensu* [20]) is narrower than the posterior edge. The terminal surfaces are not as heavily ossified as the surface of the shaft but the posterior edge is clearly longer than the anterior edge such that the ulna had an olecranon process of sorts.

BP/1/7325 is an incomplete limb bone (Fig. 25G–I) that we identify as the proximal end of a radius on the basis of its similarity to that element of the holotype of *E. africanus* [20], [74] and of *S. shansisuchus* ([17]: fig. 28c, possibly upside down). The shaft is oval in transverse section, expanded transversely and compressed dorsoventrally, and tapers slightly distally, the proximal end suboval, the distal end subcircular. On the basis of comparison with *E. africanus*, one surface (ventral *sensu* [20]) of the proximal surface in proximal view is flat to concave and the other (dorsal) convex.

#### Pelvic girdle

**Figure 27:**
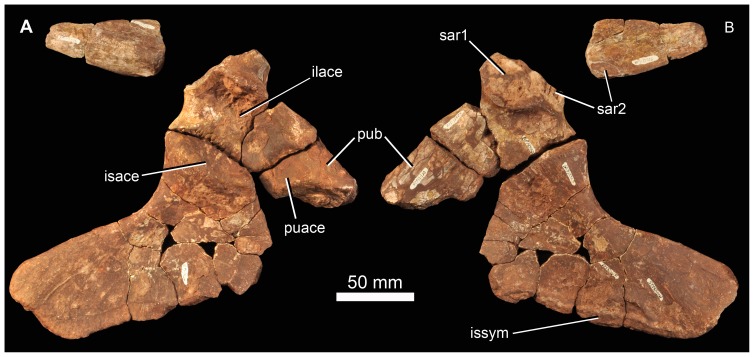
Pelvis of *Garjainia madiba* sp. nov. BP/1/5525, right pelvis in lateral (A) and medial (B) views. Abbreviations: ilace, iliac contribution to acetabulum; isace, ischial contribution to acetabulum; issym, ischial symphysis; puace, pubic contribution to acetabulum; pub, pubis; sar1, sar2, areas of articulation with first and second sacral ribs.

The ilium is represented in BP/1/5525 by an almost complete left element (missing the preactabular process of the dorsal blade; dorsal margin of blade damaged along its length; ventral margin of dorsal blade damaged at the point at which the second sacral rib contacted the base of the postacetabular process: Fig. 26A–B), parts of the right element (acetabulum including crest and posterior end of postacetabular process of dorsal blade: Figs. 26C, 27), and a fragment (supraacetabular rim) of a second right ilium. At least one other partial ilium is known (BP/1/7333, left acetabular fragment and broken postacetabular process of dorsal blade: Fig. 26D–F). The most striking feature of the ilium is the exceptionally long postacetabular process of the dorsal blade, approximately as long as (or slightly longer than) the maximum length of the acetabular part of the ilium. In this respect it differs from the corresponding element of *E. africanus*, *S. shansisuchus*, and *G. prima* and thus represents a putative autapomorphy of the new species. This postacetabular process has a convex lateral surface towards its posterior end and its ventral margin is folded inwards (deeper transversely on ventral edge than dorsal edge). The dorsal margin of the process appears to be slightly curved upwards towards its posterior end (BP/1/7333). The supraacetabular rim is prominent, anteriorly and dorsally (not posterodorsally). As in *E. africanus* and *S. shansisuchus* the surface for articulation with the pubis is shorter than that for the ischium, and the latter surface extends a substantial distance posterior to the strong waist between the dorsal blade and acetabular region of the ilium. Medially there are two large scars for the sacral ribs that are clearly delimited by ridges; the first is medial to the acetabulum and supraacetabular rim, the second is medial to the base of the postacetabular process. The second rib facet has an arched ridge close to its dorsal margin that fits into a groove on the lateral margin of the sacral rib. The moderately downturned sacral ribs means that the acetabulum faced ventrolaterally.

**Figure 28:**
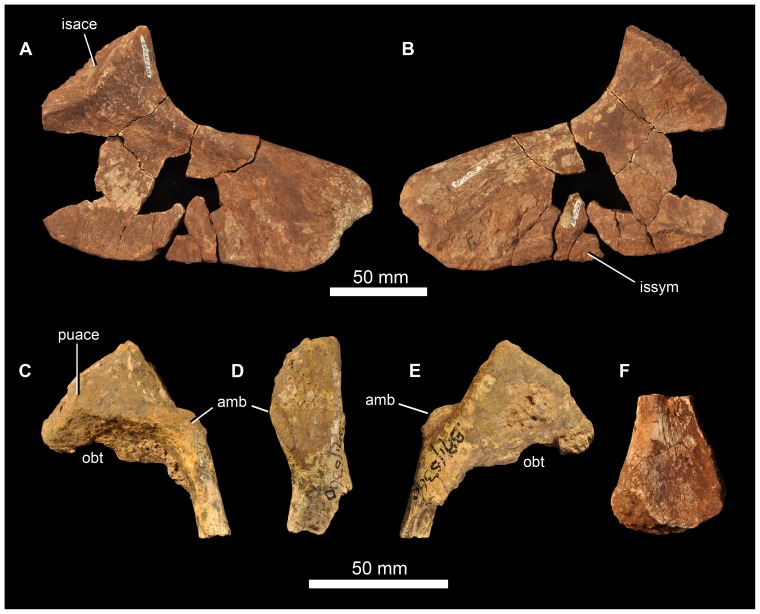
Ischium and pubis of *Garjainia madiba* sp. nov. A–B, BP/1/5525, left ischium in lateral (A) and medial (B) views. C–E, BP/1/7328, proximal right pubis in lateral (C), anterior (D) and medial (E) views. F, BP/1/5525, distal left pubis in posteroventral view. Abbreviations: amb, ambiens process; isace, ischial contribution to acetabulum; issym, ischial symphysis; obt, obturator foramen; puace, pubic contribution to acetabulum.

**Figure 29:**
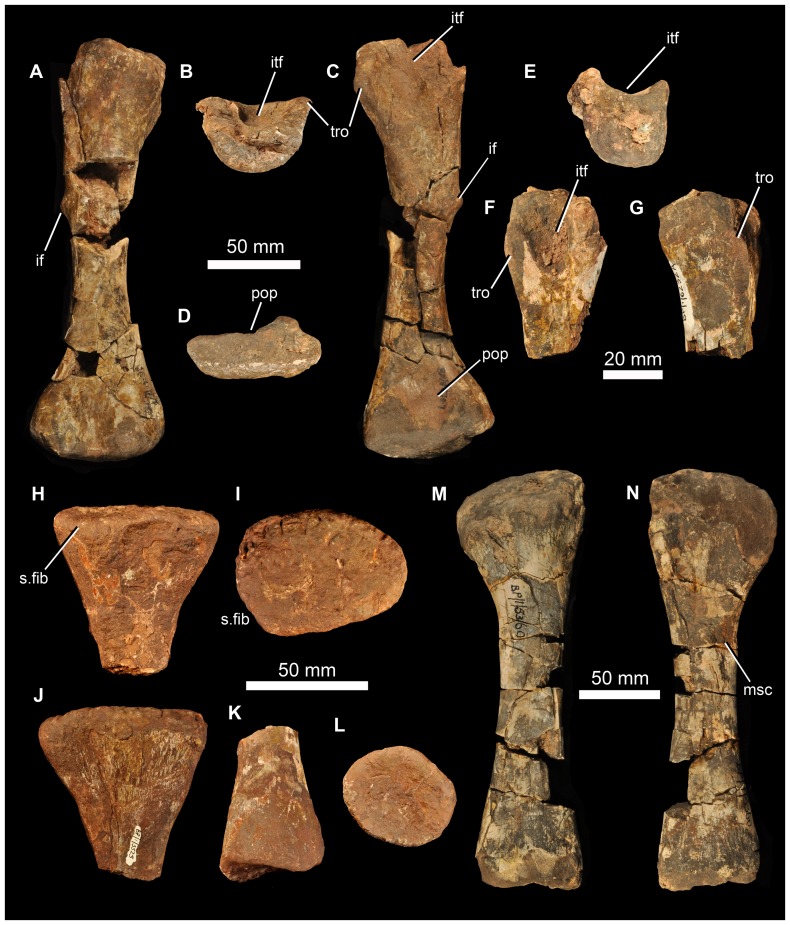
Hindlimb elements of *Garjainia madiba* sp. nov. A–D, BP/1/5767, right femur in dorsal (A), proximal (B), ventral (C) and distal (D) views. E–G, BP/1/6232a, fragmentary proximal right femur in proximal (E), ventral (F) and medial (G) views. H–J, BP/1/5525, proximal left tibia in dorsal (H), proximal (I) and ventral (J) views. K–L, BP/1/5525, distal tibia in side (K) and distal (L) views. M–N, two views of probable tibia BP/1/7341. Abbreviations: if, trochanter for attachment of *m. iliofemoralis*, itf, intertrochanteric fossa; msc, muscle scar; pop, popliteal space; s.fib, surface for articulation with fibula; tro, internal trochanter.

**Figure 30:**
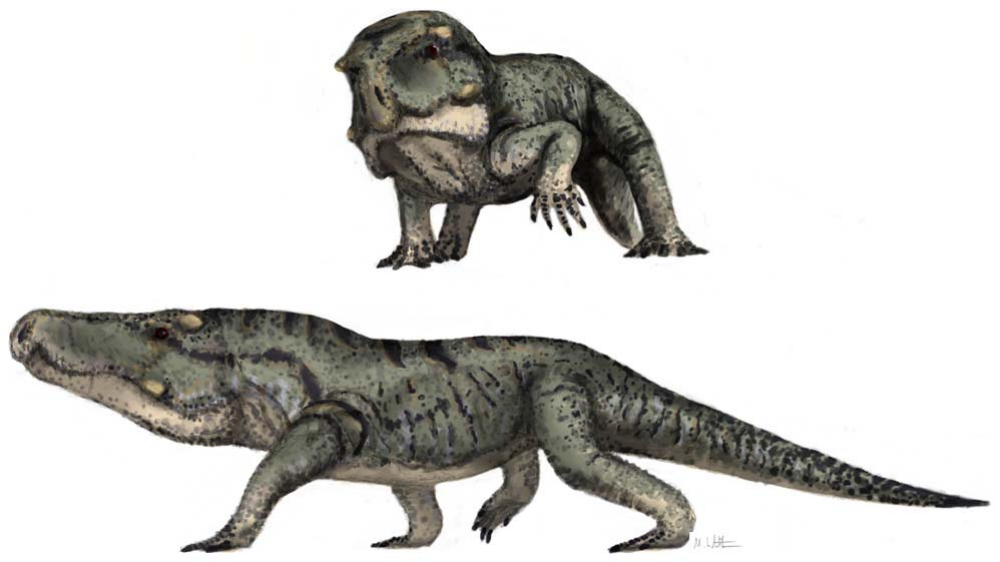
Life reconstruction of *Garjainia madiba* sp. nov. Total adult body length would have been approximately 2.5 metres, based on comparisons with *G. prima*. Reconstruction by Mark Witton.

**Figure 31:**
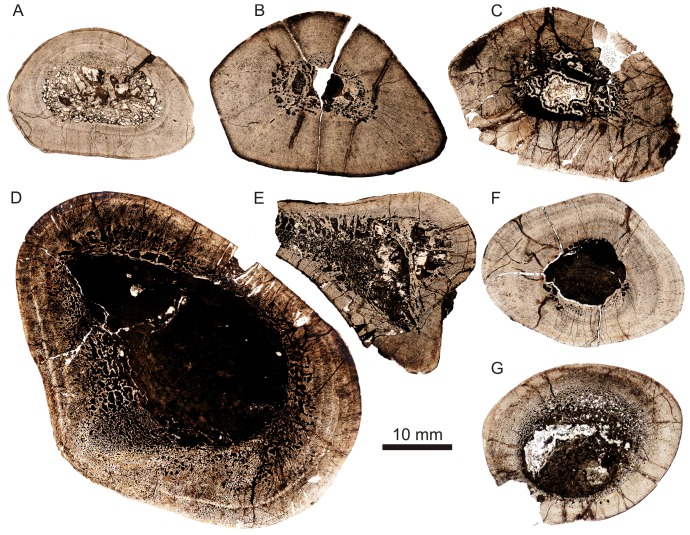
Bone histology of *Garjainia madiba* sp. nov. Transverse thin-sections of limb bones, showing relatively thick cortices and partially infilled or clear medullary cavities. A, BP/1/7129 humerus shaft. B, BP/1/7219 humerus shaft. C, BP/1/7217 distal end of femur shaft. D, BP/1/6232j femur shaft. E, BP/1/7216 proximal end of shaft of tibia. F, BP/1/7218 midshaft region of radius or tibia. G, BP/1/7130 distal end of shaft of tibia.

The ischia are well represented in BP/1/5525 (Figs. 27, 28), being almost complete on the right and 75% complete on the left. The ischium is similar to that of *E. africanus* and *G. prima*, with a straight ventral margin in lateral/medial view, unlike the W-shaped edge in *S. shansisuchus* [17]. Anterodorsally the ischium is expanded to form an articular surface for the ilium and the ischial contribution to the acetabulum. The remainder of the element is plate-like and flattened in the dorsomedial to ventrolateral plane. This plate-like part is thickened dorsally, at its ventromedial margin (where it articulated with its antimere) and at the posterior end.

Poorly preserved partial pubes are preserved in BP/1/5525, including at least the acetabular portion of the right pubis (Fig. 27) and laterodistal end of the pubic apron of the left (Fig. 28F). A better proximal right pubis is preserved in BP/1/7328 (Fig. 28C–E). The proximal end is transversely thickened into a triangular area that articulates with the ilium. Lateral to this, the pubic contribution to the anteroventral part of acetabulum is relatively minor. There is a well-developed curved flange of bone on the proximal end, anteroventral to the acetabulum, which represents the ambiens process. This process resembles that of *G. prima* (e.g., PIN 951/25), with both being notably more differentiated than the only slightly raised, roughened homologous region in *E. africanus* and *S. shansisuchus* [17], [20]. Only the anterior margin of the obturator foramen is preserved (Fig. 28C, E). The distal end of the pubis is like that of *E. africanus, S. shansisuchus* and *G. prima* in being L-shaped in transverse section, comprising a lateral rod and medial sheet.

#### Femur

The femur is relatively well known from an almost complete but fragmented right example (BP/1/5767: Fig. 29A–D), three right subproximal ends (BP/1/6232a: Fig. 29E–G, 6232j and an unnumbered specimen) and a distal right end (BP/1/5525). The femur of *G. madiba* is very similar to that of *G. prima* (see [9]: fig. 8.5C). It is relatively straight (for an archosauriform femur) in dorsal and ventral views, and there is a large proximal intertrochanteric fossa and a proximally positioned, approximately blade-like internal trochanter. The internal trochanter is positioned further proximally on the ossified part of the femur than in *E. africanus*, confluent with, but its apex angled away from, the proximal end (similar to the rhynchosaur *Hyperodapedon gordoni* as figured by [76]). Further distal to the intertrochanteric fossa, the lateral (*sensu* [18], [20]) edge bears a trochanter probably for insertion of the iliofemoralis muscle. Distally there is a shallow popliteal space and intercondylar groove, and the distal end is similar to that of the femur of *G. prima* ([23]: plate 15, fig. 2) and *E. africanus* ([20]: fig. 34E). As with *G. prima* and *E. africanus*, the proximal and distal terminal ends of the femur are incompletely ossified and would probably have been formed by cartilage in life.

#### Tibia and fibula

The proximal and distal ends of a tibia are preserved in BP/1/5525 (Fig. 29H–L). By comparison with *E. africanus* (BMNH R3592, right tibia figured by [20]: fig. 35) the proximal end is from the left side (based on the outline of the proximal surface). The proximal end is strongly expanded relative to the shaft and has a rugose proximal surface. The proximal end has an asymmetric outline, expanded laterally and tapering medially. There is no indication of a well-developed cnemial crest. The preserved fragment is not long enough to preserve the pit for the *m. puboischiotibialis* (though this feature is visible at the distal end of the proximal tibia BP/1/7216). The distal end (possibly not from the same individual bone or side as the proximal end) has a concave end surface and is identified on the basis of similarity with other erythrosuchids in terms of the subcircular shape of the distal end, degree of expansion from shaft, and dissimilarity to other epipodials (for example, the distal end of the ulna is not concave). The shaft is mostly missing, but where the proximal and distal ends are broken it has a subcircular cross section.

A complete but crushed BP/1/7341 epipodial (Fig. 29M–N) is identified as a tibia on the basis that it differs to the ulna and radius and its ends are likely too expanded to be a fibula. The proximal end is strongly expanded, with a suboval terminus, the distal end is less expanded and has an oval and concave end surface. The shaft has an oval cross section. There is a slightly depressed, roughened area (possible muscle-attachment scar) on the ventral surface of the shaft, just proximal to midlength (Fig. 29N: msc).

#### Osteoderms

There are no unambiguous osteoderms among the preserved material. Given the substantial number of archosauromorph specimens known from Subzone A of the *Cynognathus* Assemblage Zone it is likely that osteoderms were absent in *G. madiba*, but more complete associated material is required to test this further.

#### Reconstruction

An artist's impression of *G. madiba* in life is shown in Fig. 30. This is based on all of the available (type and referred) specimens and is most strongly influenced by photographs and drawings of a mounted, partly reconstructed skeleton of *G. prima* (and the almost complete holotype skull of that species) previously displayed at PIN.

### Bone Histology

All the examined elements contain large medullary cavities and a compact cortex, regardless of size (Fig. 31A–G). The primary cortex comprises a woven-fibred bone matrix that contains haphazardly arranged, loosely packed collagen fibres and numerous irregularly distributed globular osteocyte lacunae. This bone matrix is “associated with a lamellar matrix of primary osteons” and thus falls within the fibro-lamellar complex as defined by [31]. Fibro-lamellar bone has been found to be associated with rapid growth rates in extant vertebrates [35]–[36], [77]. All examined elements are highly vascularized (10.9–27.3%, Table 1). Vascular orientation varies from laminar to reticular to radial, depending on the element. Most of the elements contain growth marks that represent a temporary decrease or cessation in growth rate [31], [34] and which are typically annual [78].

#### Propodials

**Figure 32:**
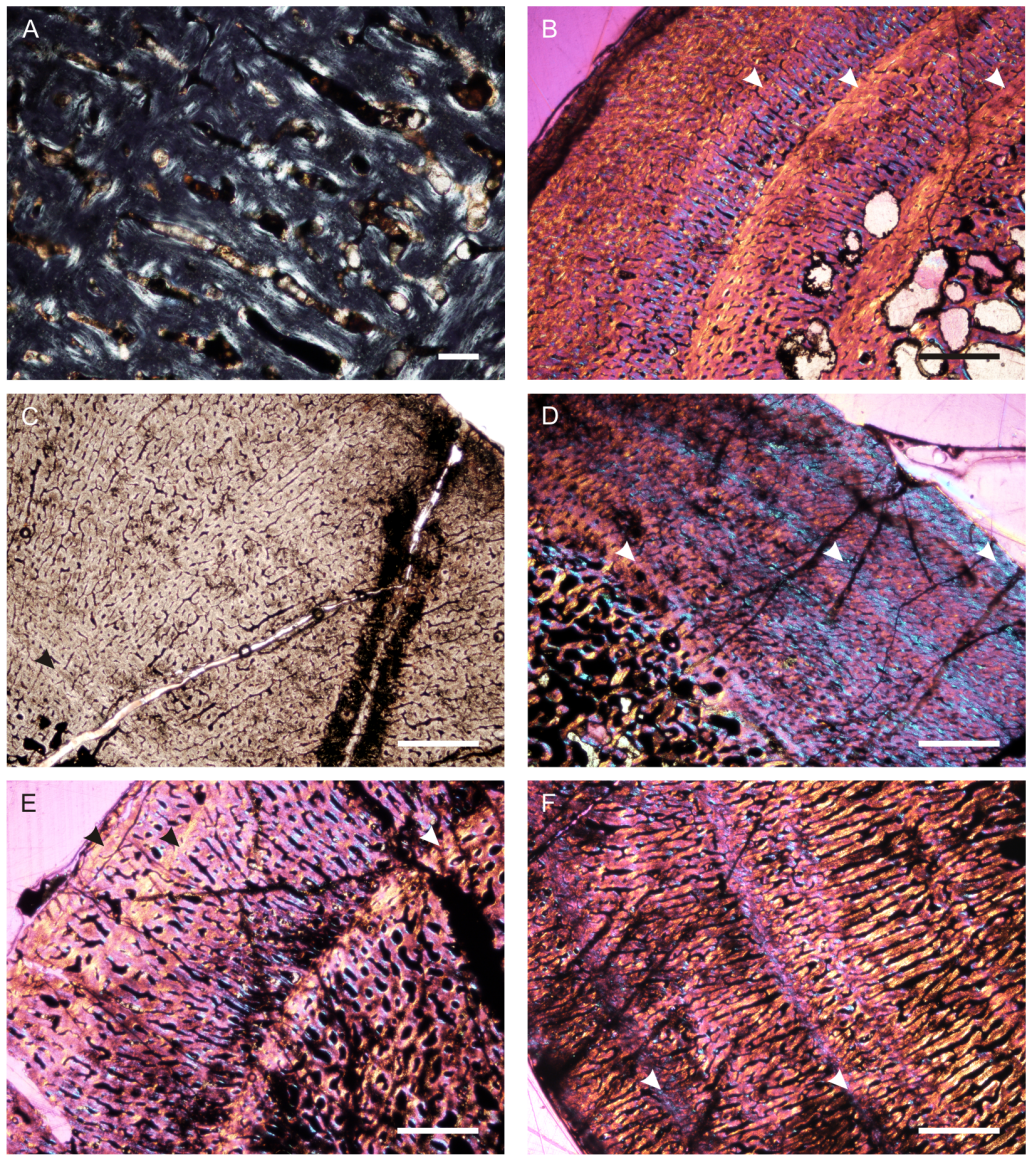
Bone histology of *Garjainia madiba* sp. nov. A, humerus BP/1/7129 showing woven-fibred interstitial matrix with abundant longitudinally-oriented and radial primary osteons under high magnification polarized light. B, humerus BP/1/7129 in cross-polarized light showing highly vascularized fibro-lamellar bone interrupted by three annuli (arrowheads) of parallel-fibred bone. C, humerus BP/1/7219 showing an annulus in the inner cortex (arrowhead) and a mixture of radial and reticular fibro-lamellar bone. Femora BP/1/7217 (D) and BP/1/6232j (E) showing broad annuli (arrowheads) of parallel-fibred bone under cross-polarized light, traversing highly vascularized fibro-lamellar bone. F, femur BP/1/6232j under cross-polarized light showing abundant radially-oriented vascular canals. Scale bars: A, 100 µm; B–F 1000 µm.

The propodial elements examined include two humeri (BP/1/7129, BP/1/7219; Fig. 31A, B) and two femora (BP/1/7217, BP/1/6232j; Fig. 31C, D). The humeri were sectioned approximately midshaft, BP/1/7219 somewhat more distally. Both have small medullary cavities that are surrounded by numerous resorption cavities, bordered by relatively thick cortices (average k  = 0.35, Table 1). Both cortices are highly compact with an average compactness of 0.838 (Table 1). The large erosion cavities have partially resorbed the innermost growth marks in both humeri. Small secondary osteons (as defined by erosional lacunae delimited by a cementing line: [31]) are present in the remodelled perimedullary regions, but are relatively rare. The primary cortex comprises highly vascularized fibro-lamellar bone tissue with abundant, globular, irregularly distributed osteocyte lacunae in a woven-fibred bone matrix (Fig. 32A), indicating rapid rates of bone deposition. The vascular canal orientation in humerus BP/1/7129 (Fig. 32B) comprises a mixed arrangement of reticular and radial canals, whereas humerus BP/1/7219 is comprised almost entirely of radiating vascular canals (Fig. 32C), with only small reticular patches. Experiments on extant vertebrates have shown that radially oriented vascular canals within a fibro-lamellar complex represent the highest rates of growth [77]. Three annuli (indicating a temporary decrease in growth rate) containing relatively slowly-forming parallel-fibred bone are preserved in BP/1/7129 and two annuli are preserved in BP/1/7219, the innermost one of the former and both of the latter being partially resorbed by secondary remodelling in the perimedullary region. Growth marks are absent from the outer cortex, and an external fundamental system (EFS), indicating a termination in growth and thus somatic maturity [80], was not observed in either element. Considering the continued high vascular and osteocyte lacuna density that extends to the subperiosteal surface, it is clear that both elements were still actively growing at the time of death. Using the distance between successive growth marks and a Triassic year of 380 days [81]–[82], bone deposition rates for the humeri are estimated to range from 9 to 18 µm/day for BP/1/7129 and 22.3 to 44.6 µm/day for BP/1/7219 (the lower value representing the most conservative estimate of active growth in one year and the higher value representing active growth during the favourable growing season, i.e. over a six month period).

The femora were sectioned in the shaft region, BP/1/6232j approximately midshaft and BP/1/7217 more distally. Both contain large free medullary cavities that are surrounded by numerous erosion cavities (Fig. 31C, D). Bone drift is prominent in the larger femur, BP/1/6232j, in the area of the adductor crest (for attachment of *m. adductor femoralis*), with large resorption cavities indicating an area of higher localized strain (Fig. 31D). Femur BP/1/7217 has a similar compactness value to the humeri (C = 0.812), but the larger femur, BP/1/6232J, has a relatively lower value of 0.489, indicating a higher porosity compared to femur BP/1/7217 and the humeri. The femora have slightly thinner cortices compared to the humeri (average k  = 0.5, Table 1). Their bone tissues are comprised of highly vascularized, rapidly forming fibro-lamellar bone interrupted by faint annuli (Fig. 32D, E). The vascular canals in both femora form a mixture of reticular and radial arrangements (average POD 193 µm), the latter being more prominent in the larger femur BP/1/6232j (Fig. 32F). The annuli are not particularly distinct and contain a poorly vascularized bone tissue of parallel-fibred bone. The innermost annuli are partially resorbed in both elements. Both femora lack an EFS. Bone deposition rates are estimated to range from 10.3 to 20.6 µm/day for BP/1/7217 and 6.6 to 13.2 µm/day for BP/1/6232j.

#### Epipodials

**Figure 33:**
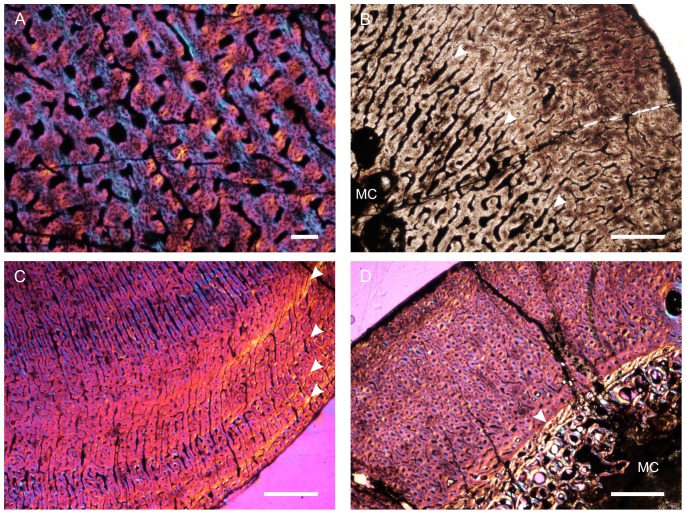
Bone histology of *Garjainia madiba* sp. nov. A, proximal tibia BP/1/7216 showing abundant vascular canals and osteocyte lacunae in a woven-fibred interstitial matrix in cross-polarized light. B, proximal tibia BP/1/7216 showing radiating vascular canals in the inner cortex bordred by a change in vascular canal orientation (arrowheads). C, radius or tibia BP/1/7218 showing a dominance of radiating vascular canals in cross-polarized light. Annuli are indicated by arrowheads. D, distal tibia BP/1/7130 showing a reversal line bordering compacted coarse cancellous bone (arrowhead) under cross-polarized light. MC, medullary cavity. Scale bars: A, C, and D 1000 µm; B 100 µm.

The sectioned epipodials comprise three elements (Fig. 31E, F, G). BP/1/7216 is the proximal end of a tibia (broken at the level of a pit for attachment of the *m. puboischiotibialis*) and BP/1/7130 is the distal end of a tibia. BP/1/7218 represents the shaft region of an epipodial (not a femur or humerus, which both have distinctive shafts) that, based on the shape of the shaft cross section and degree of expansion towards one of the preserved ends, is likely a radius or tibia (not an ulna or fibula). Among the sectioned elements, BP/1/7218 is the one that we least confidently refer to *G. madiba*, though this identification is not inconsistent with absolute size or locality. The proximal end of the shaft of BP/1/7216 (Fig. 31E) has a large, partially infilled medullary cavity that contains numerous thin cancellous bony trabeculae and several thick bony struts. Small secondary osteons are present but are limited to the perimedullary region. The bone tissues are similar to those of the propodials in comprising highly vascularized woven-fibred bone in a fibro-lamellar complex (Fig. 33A). There are no clear annuli or LAGs, but there is a distinct change in vascular density, size and orientation midway through the cortex (Fig. 33B), suggesting a change in growth rate here. The inner cortex contains large, abundant vascular canals, many of which radiate out from the medullary cavity. From the midcortex to the outer periphery, the vascular canals decrease in size and density, and form a reticular network resulting in the appearance of a band around most of the cross section. There is no EFS.

BP/1/7218 (sectioned approximately midshaft) contains a large, completely clear medullary cavity (Fig. 31F). A few large resorption cavities are present on only one side of the bone, but at least two of the inner annuli have been partially resorbed. A thick layer of endosteal circumferential lamellae partially surrounds the medullary cavity. The relatively thick (k = 0.4), compact cortex contains highly vascularized fibro-lamellar bone with predominantly radiating vascular canals (Fig. 33C). Some regions contain a reticular arrangement between annuli. At least six annuli can be seen, but an EFS is absent and the continued high vascular density at the subperiosteal surface indicates that the bone was still actively growing at the time of death (estimated bone deposition rate 4.9 to 9.8 µm/day). The annuli consist of poorly vascularized parallel-fibred bone and although they form bands around the cortex, are relatively narrow and indistinct.

The section through BP/1/7130 (Fig. 31G) has a large medullary cavity partially infilled by bony trabeculae. The cortex is relatively narrow because the section was taken from the distal metaphysis and not the midshaft. Growth rings are not observed, but the commencement of compacted, coarse cancellous bone bordered by a reversal line on one side of the bone (Fig. 33D), the metaphyseal region from where the section was taken, and the frequency of annuli being present only in the inner cortex of these elements, indicates that the possibility of inner annuli being resorbed (as seen by the partial resorption of annuli in the inner cortex in the other elements) cannot be excluded. No deposition rate was estimated for this element because the thin section was taken from the distal metaphysis where the thinner primary cortex would cause the bone deposition rate to be underestimated.

The bone tissues of these epipodials are similar to those of *Erythrosuchus africanus* (see fibula documented by [83]; radius and tibia by [79]) in the presence of highly vascularized fibro-lamellar bone. Differences include the predominance of radiating vascular canals in most elements of *G. madiba*, compared to a predominantly reticular network in *E. africanus*, and the presence of annuli in *G. madiba*, indicating seasonal variation in growth, even during early ontogeny.

## Discussion

### Diagnosis of Erythrosuchidae

Diagnoses for Erythrosuchidae or any of its constituent genera and species are not well established. The recent phylogenetic definition of Erythrosuchidae [52] has not greatly advanced a morphological diagnosis because it remains unclear which taxa fall within this clade (many taxa and characters have yet to be included in explicit phylogenetic analyses) and the morphology of several Early Triassic non-crown-group archosauromorphs is not yet adequately documented [10]. Here we offer a diagnosis for the least inclusive clade including *Erythrosuchus*, *Garjainia* and *Shansisuchus* – the genera that comprise the core (perhaps all) of Erythrosuchidae. This diagnosis includes a combination of plesio- and apomorphic features.

Erythrosuchidae comprises archosauriforms with the following features (* probably plesiomorphic for Archosauriformes; ** probably apomorphic within Archosauriformes; *** potential autapomorphies for Erythrosuchidae): *intercentra; *laterosphenoid; *lateral mandibular fenestra; *mesotarsal joint with laterally directed calcaneal tuber; *absence of osteoderms; *premaxilla not downturned *sensu* [69] (assuming a strongly downturned premaxilla is not ancestral for Archosauriformes); **absence of ossified astragalocalcaneal canal; **absence of distal tarsals 1 and 2; **absence of tarsal centrale; metatarsal 3 longer than metatarsal 4; **somewhat verticalized parabasisphenoid (*sensu* [54]); **absence of teeth on at least the palatal ramus of the pterygoid; ***substantial ventral tongue of nasal interjecting between premaxilla and maxilla between naris and antoribital fenestra; ***dentary with three posterior processes, central one of which partly articulates with medial surface of surangular; ***long deltopectoral crest on humerus (though not quite as long as the 38% of the total humeral length reported by [52]); ***external foramen for cranial nerve VI entirely within prootic (though present also in at least the poposauroid archosaurs *Xilousuchus sapingensis*: [54], and *Arizonasaurus babbitti*: [84]); ***surface on ilium for articulation with ischium extends notably behind supraacetabular waist of ilium [52].

The state of many of the characters listed above is not yet known in several early archosauriform taxa but this proposed diagnosis would exclude *Fugusuchus hejiapensis* and *Sarmatosuchus otschevi* from Erythrosuchidae; these taxa were considered to be proterosuchids by Gower & Sennikov [9], [69] though this view is not held universally (see [10]). The diagnosis is neutral regarding the possible inclusion of several poorly known, potential erythrosuchids such as *Guchengosuchus shiguaiensis* and *Youngosuchus sinensis*.

Ezcurra et al. ([52]: 1442) listed additional character states purported to be erythrosuchid synapomorphies but which we are less convinced by: elliptical infratemporal fenestra (we are unconvinced that erythrosuchids have a clearly differently shaped lower temporal fenestra and comparative data are scant); supraoccipital excluded from border of foramen magnum by contact between opposite exoccipitals (see [54]: 900); absence of palatal teeth (some palatal teeth present in at least *G. prima*: [20]); short anterior vertebrae (also in the putative proterosuchid *S. otschevi* but not in the possible erythrosuchid *Guchengosuchus shiguaiensis*); middorsal prezygapophyses anterodorsally oriented (not thoroughly checked among early archosauromorphs in comparative approach by us); presence of pineal fossa (possibly absent in *Shansisuchus*); first sacral rib plate-like (unclearly described character and character state, not thoroughly checked among early archosauromorphs in comparative approach by us).

Parrish [85] proposed eight synapomorphies of Erythrosuchidae. Although some of these character states might emerge as diagnostic when knowledge of archosauriform anatomy and phylogeny improves, six of these we also do not find yet compelling: deeply excavated presacral centra (character not clearly optimized for archosauriforms; condition not documented for most proterosuchids; character state present in at least some other Early and Middle Triassic archosauriforms, e.g., *Arizonasaurus babbitti*: [86]; *Xilousuchus sapingensis*: [7]); absence of palatal teeth (not in *G. prima*: [20]); convex ventral margin of maxilla (present in other early archosauriforms including *Fugusuchus hejiapensis*: [65]; *Arizonasaurus babbitti*: [86]; *Xilousuchus sapingensis*: [7]); prominent lateral and medial processes of basioccipital (the ‘lateral’ processes are misidentified, large ventral rami of the opisthotics: [54]); prominent posterior ridge between tubera of parabasisphenoid and basioccipital (much wider distribution among Archosauromorpha: [54]); nearly circular coracoid not longer than anteroposterior length of scapula (not true for *G. prima*: [9]: fig. 8.5). At least two of Parrish's [85] proposed synapomorphies demand closer reassessment, and with more anatomical data, improved phylogenetic hypotheses and careful character state definitions they might prove to be diagnostic for erythrosuchids: premaxilla-maxilla ‘step’ ( =  notch); deep process-in-notch articulations between postorbital-squamosal and jugal-quadratojugal.

### Identification and Relationships of *Garjainia madiba*

Conservatism in referring multiple specimens to a single species, where these specimens do not overlap anatomically and display diagnostic apomorphies, is related to a desire to avoid spurious faunal correlations based on overly liberal and precise identifications of fragmentary material (e.g., [87]). We have been less conservative here than some workers would advocate because of the lack of evidence for more than one erythrosuchid in Subzone A of the *Cynognathus* Assemblage Zone, and the great overall similarity of the specimens we refer to *G. madiba* to the known material of *G. prima*. The decision to refer all the South African and the Russian *Garjainia* specimens to *G. madiba* and *G. prima*, respectively, is based to some degree (especially for the more fragmentary specimens) on the assumption that the two species did not overlap (spatially and perhaps also temporally) with respect to the fossil deposits from which they are currently known. Further discoveries and analyses of fossils can test our taxonomic hypothesis.

The size range of the material we refer to *G. madiba* is greater than for the known material of *G. prima*. At first glance, some of the *G. madiba* material appears to be more massive and robust than for homologous elements of its conspecific, but this is restricted mostly to some of the larger isolated vertebrae, and articular and retroarticular regions of the lower jaw, and specimen incompleteness makes it difficult to separate absolute size from shape in these comparisons.

When *Garjainia madiba* is scored for the 35 characters presented by Gower & Sennikov [54], [69], its row in a data matrix would read?00?1??1??01?10???00?00?1?1?0??00?2 in comparison with the 100111111101110??100100011110100012 for *G. prima*. Thus, there are no scoring differences other than missing data (“?”), and these are distributed in a way such that *G. madiba* can not be recovered in a more parsimonious position (see [88]) than as the sister to *G. prima* when added to Gower & Sennikov's [54], [69] data and analysed in the same way as in those studies.

### Function of Bosses on the Skull of *Garjainia madiba*

What is the function of the bosses on the lateral surface of the skull of *G. madiba*? The position of the bosses above and below the orbit might have offered some protection to the eye. However, the only preserved dorsal end of a quadratojugal of *G. madiba* indicates that there might be a thickening here also. Without more material of *G. madiba* it is not possible to address intraspecific variation with respect to ontogeny or sex that would allow, for example, testing of the hypothesis that they are sexually dimorphic and potentially involved in intraspecific communication associated with reproduction. Unusual structures in extinct organisms can be difficult to interpret, particularly without large samples of associated elements (e.g. [89]–[91]). The known material of *G. prima* has lateral ridges on the postorbital and jugal in similar positions to the much more substantial and hemispherical bosses of *G. madiba*, and this suggests that the clearly apomorphic condition in *G. madiba* is likely an enhancement of an ancestral condition rather than the acquisition of an entirely novel feature. The smaller jugal bosses among the known material of *G. madiba* appear to have all been borne by smaller jugals, but the specimens are too isolated and incomplete to assess this in more detail and we lack direct measures of the maturity of these animals.

### Bone Histology

All sectioned *Garjainia madiba* elements have a relatively thick, compact cortex, with a mean compactness value of 0.744 and k varying from 0.2 to 0.57 (Table 1). The mean cortical thickness (0.42) falls within the ranges of other non-archosaurian archosauriforms such as *Proterosuchus* (mean k  =  0.57), *Euparkeria* (0.538) and *Erythrosuchus* (0.22) [79], and bone compactness in *G. madiba* is most similar to that of *E. africanus* (C = 0.856 for the radius of the latter, [79]). Relatively thick cortices have been interpreted as indicative of an aquatic or amphibious lifestyle because the thick bone wall would counteract positive buoyancy in the water [44], [92]–[94]. Extant crocodilians, for example, have notably thick cortices (k = 0.22, [95]). In extreme cases, the bones of fully aquatic animals may also exhibit broad transition zones where the cortex transforms gradually into a medullary cavity that may be completely infilled by cancellous trabeculae [42]. However, large animals also tend to have notably thick cortices because they are stronger under localized impact than thinner bone walls [38]. Given their large body sizes (> 2 m) and lack of any unambiguous aquatic morphological or histological features, we find no evidence to challenge the interpretation that both *E. africanus* and *G. madiba* were largely terrestrial. *Garjainia madiba* has been found in, but not exclusively, fluviolacustrine deposits (see Geological Setting section), but there are no anatomical data to indicate an aquatic habitus.

The bone tissues of all *G. madiba* elements examined consist of a woven-fibred interstitial matrix dominated by large abundant primary osteons, indicating rapid growth rates [36], [77]. Comparing bone growth rates with other archosauriforms is difficult because estimates vary among elements and through ontogeny. Further research on extant taxa is required to accurately quantify bone deposition rates in extinct taxa. That said, there have been several attempts in the literature to broadly estimate growth rates in order to make interspecific comparisons. For example, Montes et al. [96] estimated that the last common ancestor of Archosauria had a bone deposition rate of approximately 13.5 µm/day, and Botha-Brink & Smith [79] estimated rates of 11.3 to 22.6 µm/day in *Proterosuchus fergusi* and 21 to 42 µm/day in *Erythrosuchus africanus*. Mean bone deposition rate estimated for the *G. madiba* limb bones in this study ranges from 9.8 to 19.5 µm/day, with a maximum of 45 µm/day, which is broadly similar to the estimates for other non-archosaurian archosauriforms [79], [97]. The rapidly forming fibro-lamellar bone in *G. madiba* is similar to the bone tissues of many other Early and Middle Triassic archosauromorphs, at least during early ontogeny [79], [83]. However, the growth of *G. madiba* differs from other non-archosaurian archosauriforms such as *Chanaresuchus* sp., *P. fergusi* and *Euparkeria capensis* [79], [83] in continuing to exhibit highly vascularized fibro-lamellar bone even towards the upper end of its known size range (and thus likely late ontogeny). In contrast, the bone tissues of *C.* sp., *P. fergusi* and *E. capensis* exhibit slowly forming lamellar-zonal bone during what has been interpreted as mid and late ontogeny [79], [83]. This difference will require reassessment if substantially larger *G. madiba* material is discovered.

Overall, the bone histology of *G. madiba* is most similar to that of the South African erythrosuchid *Erythrosuchus africanus* from *Cynognathus* Subzone B, but differs in the presence of annuli in the limb bones, even during early ontogeny. To date, annuli, indicating a temporary decrease in growth rate, have not been found in any limb bones of *E. africanus* [79], [83], [97]–[98], but their presence in ribs and girdle fragments [79] indicate nonetheless that *E. africanus* was capable of variable growth. Given its close relationship with *G. madiba* and the fact that relatively few limb bones of *E. africanus* have been sectioned and that many of them are badly preserved, the absence of limb bone growth rings in the latter taxon requires further testing.

It is worth noting that many *G. madiba* elements contain annuli only in the inner cortex and/or exhibit thick regions of rapidly growing, radiating fibro-lamellar bone tissue between the inner and outermost annuli (e.g. Fig. 31A, B, E, G), indicating bursts of rapid growth and a substantial amount of bone deposition during the active growing season(s). These inferred growth spurts may have enabled this species to reach a large body size relatively quickly, and they support suggestions that high growth rates might have been achieved prior to the origin of ornithodiran archosaurs [79], [83], [95], [99]–[101].

### Evolutionary, Biogeographic and Biostratigrapic Implications

*Garjainia madiba* represents the geologically oldest known erythrosuchid from the Southern Hemisphere, having been recovered from a level that is demonstrably stratigraphically lower than classic lower Middle Triassic occurrences of *Erythrosuchus africanus* in South Africa. The shared presence of the mastodonsaurid temnospondyl genus *Parotosuchus* in the *Cynognathus* Assemblage Zone Subzone A of South Africa, the Middle Buntsandstein of Germany and the Yarenskian Gorizont of Russia [102] provides biostratigraphic evidence that Subzone A is late Olenekian (late Early Triassic) in age (e.g. [22], [59], [103]). The shared presence of *Garjainia* in the Yarenskian Gorizont and *Cynognathus* Assemblage Zone Subzone A has also been used to support this correlation [22], [59], although the strength of this evidence depends on at least the monophyly of *Garjainia*. If the late Olenekian age for Subzone A is correct, then *G. madiba* is approximately contemporaneous with the geologically oldest erythrosuchid records from the Northern Hemisphere. Together with these Northern Hemisphere records *G. madiba* indicates that erythrosuchids became established as the largest terrestrial predators across a broad swath of Pangaea within five million years of the end-Permian mass extinction event.

Three very different local tetrapod ‘faunas’ are known from the late Olenekian of Eastern Europe and the Cis-Urals, each restricted to the more northern (central and northern regions of the East European Platform and the Timan-North Ural region), southwestern (southern East European Platform) and southeastern (southeastern East European Platform and southern Cis-Urals) regions [27], [104]. Only the southeasternmost of these ‘faunas’ shares closely similar elements with Gondwana, including the temnospondyl *Rhytidosteus* and now also the erythrosuchid *Garjainia*.

The archosauromorph faunal assemblage of *Cynognathus* Assemblage Zone Subzone A provides an important temporal link within southern Pangaea between the *Lystrosaurus* Assemblage Zone (earliest Triassic), the archosauromorph component of which is represented by *Proterosuchus*, *Prolacerta* and an unnamed species [105], and *Cynognathus* Assemblage Zone Subzone B (early Middle Triassic), which includes *Erythosuchus*, *Euparkeria*, *Howesia* and *Mesosuchus*. Archosauromorph material from Subzone A remains incompletely studied, but has substantial potential for yielding new insights into the establishment of typical Triassic archosauromorph-dominated ecosystems.

## Supporting Information

Text S1 List of comparative proterosuchian material, and list of known material of *Garjainia madiba* sp. nov.
(DOCX)
