# Supplementary material for: A New Species of Garjainia Ochev, 1958 (Diapsida: Archosauriformes: Erythrosuchidae) from the Early Triassic of South Africa
Source: PLoS One. 2014 Nov 11;9(11):e111154. doi: 10.1371/journal.pone.0111154 (PMC4227673; doi:10.1371/journal.pone.0111154)
Supplement: Text S1 — List of comparative proterosuchian material, and list of known material of Garjainia madiba sp. nov. (DOCX) [file pone.0111154.s001.docx]

SUPPLEMENT TO:

**A New Species of *Garjainia* Ochev, 1958 (Diapsida: Archosauriformes: Erythrosuchidae) from the Early Triassic of South Africa**

**David J. Gower^1,*^, P. John Hancox^2^, Jennifer Botha-Brink^3^, Andrey G. Sennikov^4,5^, Richard J. Butler^2,6,7^**

**1** Department of Life Sciences, The Natural History Museum, London SW7 5BD, UK, **2** Evolutionary Studies Institute, University of the Witwatersrand, Private Bag 3, Wits 2050, South Africa, **3** Karoo Palaeontology, National Museum, PO Box 266, Bloemfontein 9300, South Africa and Department of Zoology and Entomology, University of the Free State, Bloemfontein, 9300, South Africa, **4** Borissiak Paleontological Institute RAS, Profsoyuznaya 123, Moscow 117647, Russia, **5** Kazan Federal University, Kremlyovskaya 18, 420008, Kazan, Russia, **6** School of Geography, Earth and Environmental Sciences, University of Birmingham, Edgbaston, Birmingham B15 2TT, UK, **7** GeoBio-Center, Ludwig-Maximilians-Universität München, Richard-Wagner-Straße 10, D-80333 München, Germany

**1. COMPARATIVE PROTEROSUCHIAN Material**

***Garjainia prima*** Ochev, 1958 (including its subjective junior synonym *Vjushkovia triplicostata* (Huene, 1960)

Personal observation (DJG, AGS, RJB) of the type specimens of *G. prima* and *V. triplicostata* documented by Ochev (1958) and Huene (1960). Literature: Ochev (1958, 1975, 1981), Huene (1960), Tatarinov (1961), Charig & Reig (1970), Charig & Sues (1976), Parrish (1992), Gower (1996, 2003), Sennikov (1995, 2008), Gower & Sennikov (1996, 1997, 2000).

***Erythrosuchus africanus* Broom, 1905**

Personal observation (RJB, DJG, JH, AGS) of material documented by Gower (1996, 1997, 2003). Literature: Broom (1905, 1906), Huene (1911), Brink (1955), Hughes (1963), Charig & Reig (1970), Charig & Sues (1976), Cruickshank (1978), Parrish (1992), Juul (1994), Gower (1996, 1997, 2001, 2003), Nesbitt (2011).

***Shansisuchus shansisuchus*** Young, 1964 (including its junior synonym, *S. heiyuekouensis* Young, 1964)

Personal observation (DJG) of material documented by Young (1964). Literature: Young (1964), Charig & Reig (1970), Charig & Sues (1976), Parrish (1992), Gower (1996), Gower & Sennikov (1996).

***Shansisuchus kuyeheensis*** Cheng, 1980

Personal observation (DJG) of material documented by Cheng (1980). Literature: Cheng (1980).

***Chalishevia cothurnata*** Ochev, 1980

Personal observation (DJG, AGS, RJB) of type material documented by Ochev (1980) and nasals referred by Sennikov (1995). Literature: Ochev (1980), Sennikov (1995, 2008), Gower & Sennikov (2000).

***Uralosaurus magnus*** (Ochev, 1980)

Personal observation (DJG, AGS, RJB) of type material documented by Ochev (1980). Literature: Ochev (1980), Sennikov (1995, 2008), Gower & Sennikov (2000)

***Youngosuchus sinensis*** (Young, 1973)

Personal observation (DJG, RJB) of type material documented by Young (1973). Literature: Young (1973), Charig & Sues (1976), Kalandadze & Sennikov (1985), Parrish (1992).

***Guchengosuchus* *shiguaiensis*** Peng 1991

Personal observation (RJB) of type material documented by Peng (1991). Literature: Peng (1991)

***Fugusuchus hejiapensis*** Cheng, 1980

Personal observation (DJG) of type material documented by Cheng (1980). Literature: Cheng (1980), Parrish (1992), Gower & Sennikov (1996).

***Sarmatosuchus otschevi*** Sennikov, 1994

Personal observation (DJG, AGS, RJB) of material documented by Sennikov (1994). Literature: Sennikov (1994), Gower & Sennikov (1997, 2000).

***Proterosuchus fergusi*** Broom, 1903

Personal observation (RJB, DJG, JH) and published descriptions. Literature: Broom (1903, 1932, 1946), Haughton (1924), Broili & Schröder (1934), Brink (1955), Hughes (1963), Hoffman (1965), Charig & Reig (1970), Cruickshank (1972), Charig & Sues (1976), Sereno & Arcucci (1990), Sereno (1991), Clark et al. (1993), Juul (1994), Welman & Flemming (1993), Welman (1998), Klembara & Welman (2009), Nesbitt (2011).

**2. MATERIAL OF *GARJAINIA MADIBA* SP. NOV.**

All localities are farms in Thabo Mofutsanyana district municipality, Free State province, South Africa

**A. Holotype and paratypes**

*Driefontein 11*

BP/1/5360, assemblage of material including two partial cervical vertebrae (not figured), one dorsal centrum (not figured), partial neural spines (not figured). BP/1/5360 previously included a larger collection of mostly postcranial elements that have now been renumbered (see below). On our most recent inspection (May, 2014) we could not find all of the 5360 material and it is possible that some of the axial material still listed here under BP/1/5360 might also have been renumbered.

BP/1/5760 (holotype, Figs. 5-7, 18), Right postorbital, postfrontal, and partial frontal; ventral end of left postorbital, left jugal; right paroccipital process with partial supraoccipital and parts of right prootic in articulation; partial rib; at least four unidentified fragments.

BP/1/5760a, partial left premaxilla (Fig. 8A-C).

BP/1/5760b, partial anterior cervical vertebra (not figured)

BP/1/5763b, right articular and posterior part of surangular (Fig. 16H-K).

BP/1/5767, right femur (Fig. 28A-D).

BP/1/5769, three fused caudal vertebrae (Fig. 20I).

BP/1/6232a, proximal right femur (Fig. 28E-G).

BP/1/6232b, left articular and surangular.

BP/1/6232c, angular (Fig. 16F-G).

BP/1/6232d, partial right quadrate (Fig. 12J-K).

BP/1/6232e, partial left quadrate (not figured).

BP/1/6232f, partial left quadrate (not figured).

BP/1/6232g, isolated right jugal boss (not figured).

BP/1/6232h, isolated right jugal boss (Fig. 11I).

BP/1/6232i, isolated left jugal boss (Fig. 11J).

BP/1/6232j, partial right femur (Figs. 30D, 31E, F).

BP/1/6232k, partial right dentary (not figured).

BP/1/6232l, partial left premaxilla (Fig. 8H-I).

BP/1/6232m, partial right maxilla (Fig. 10D-F).

BP/1/6232n, partial right premaxilla (Fig. 8D-G).

BP/1/6232o, parabasisphenoid (not figured).

BP/1/6232r, right ulna (Fig. 24A-F).

BP/1/6232v, partial parabasisphenoid (not figured).

BP/1/6232w, partial parabasisphenoid (Fig. 15B).

BP/1/6232x, partial parabasisphenoid (not figured).

BP/1/6232y, partial parabasisphenoid (Figs. 13E, 14E).

BP/1/6232z, base of paroccipital process (not figured).

BP/1/6232aa, basioccipital (not figured).

BP/1/6232ac, caudal vertebra (Fig. 20C-D).

BP/1/6232ad, partial left postorbital (not figured).

BP/1/6232ae, cervical vertebra (Fig. 17J).

BP/1/6232ag, partial left postorbital (Fig. 11H).

BP/1/6232ai, cervical vertebra (not figured).

BP/1/6232aj, anterior dorsal vertebra (Fig. 18I-J).

BP/1/6232ak, dorsal vertebra (Fig. 18F-H).

BP/1/6232al, cervical vertebra (not figured).

BP/1/6232am, anterior dorsal vertebra (not figured).

BP/1/6734, right jugal boss (not figured).

BP/1/6735, right quadratojugal (Fig. 13D-E).

BP/1/6738, partial first sacral vertebra and rib (not figured)

BP/1/6791, partial left postorbital and partial ?articular (not figured)..

BP/1/7132, right postorbital, partial rib (not figured).

BP/1/7133, partial left postorbital (not figured).

BP/1/7135, dorsal vertebra (Fig. 18D-E).

BP/1/7152, right scapula (Fig. 21A-B).

BP/1/7153, anterior right dentary (Fig. 16E), tooth, articular + partial surangular (not figured).

BP/1/7325, proximal radius (Fig. 24G-I). Previously part of BP/1/5360.

BP/1/7326, posterior fragment of right surangular and articular (not figured).

BP/1/7327, isolated partial cervical vertebra (not figured).

BP/1/7328, proximal right pubis (Fig. 25C-E). Previously part of BP/1/5360.

BP/1/7329, mid/posterior caudal centrum (not figured).

BP/1/7330, fragment of midshaft of long bone (not figured). Previously part of BP/1/5360.

BP/1/7331, dorsal neural spine (not figured).

BP/1/7333, acetabular and postacetabular regions of left ilium (Fig. 25D-F). Previously part of BP/1/5360.

BP/1/7334, mid/posterior caudal vertebra (Fig. 20J). Previously part of BP/1/5360.

BP/1/7335, anterior caudal vertebra (Fig. 20E-H). Previously part of BP/1/5360.

BP/1/7336, complete right humerus (Fig. 23A-D). Previously part of BP/1/5360.

BP/1/7337, partial left postorbital (not figured). Previously part of BP/1/5360.

BP/1/7338, incomplete posterior cervical vertebra (Fig. 17G-I). Previously part of BP/1/5360.

BP/1/7339, cervical neural spine (not figured).

BP/1/7341, crushed tibia (Fig. 28M-N). Previously part of BP/1/5360.

BP/1/7342, fragment of dorsal part of braincase (not figured).

BP/1/7346, fragmentary neural spine (not figured).

*Guarriekop 330*

BP/1/5525, two partial right premaxillae, partial right maxilla (Fig. 10A-C), possible fragment of second right maxilla (not figured), ?dorsal fragment of left jugal (not figured), fragment of right frontal (Fig. 11D-E), posteroventral part of braincase (basioccipital, parabasisphenoid, exoccipital, opisthotic, prootic) including floor of endocranial cavity (Figs. 13, 14), partial angular (not figured), partial right quadrate missing midshaft (Fig. 12F-I), two sacral vertebrae and ribs (Fig. 19), many partial vertebrae and neural spines (includes at least 3 cervicals, at least 8 dorsals, at least 4 caudals, and many fragments: Figs. 17, 20), nearly complete left ilium (Fig. 25A-B), incomplete left ischium (Fig. 27A-B), partial right ilium (Fig. 25C, 26), fragment of a second right ilium (not figured), nearly complete right ischium and proximal right pubis (Fig. 26), distal left pubis (Fig. 27F), two distal femora (not figured), proximal left tibia (Fig. 28H-J), distal tibia (Fig. 28K-L), two small distal right femora. Majority of material is consistent with referral to a single individual, but some duplicate and smaller elements (premaxillae, femora) indicate presence of at least one additional individual.

BP/1/6233a, shaft of right humerus (not figured).

BP/1/6233b, dorsal vertebra (Fig. 18A-C).

BP/1/7138, poorly preserved and/or unprepared material, including several vertebrae, sacral rib, partial left postorbital, right premaxilla (not figured), and fragmentary maxillae (Fig. 10G-H).

BP/1/7342, part of roof of endocranial cavity and proximal end of left paroccipital process (Fig. 15A). Previously numbered BP/1/5525a.

*Fraaiuitsicht/Bosrand 12*

BP/1/6226a, partial right prefrontal (Fig. 11A-C).

BP/1/6226b, right jugal boss (not figured).

*Platkop*

BP/1/7215, left quadratojugal (Fig. 12A-C).

*Eerste Geuluk*

NMQR 3049, partial right dentary; posterior ends of left and right mandibles (not figured).

NMQR 3051, anterior end of left mandible (Fig. 16A-D), partial left scapula (Fig. 21C-F), partial right humerus (Fig. 23E-G), isolated teeth, partial left coracoid (Fig. 22).

*Langberg 556*

NMQR 3257, left premaxilla (Fig. 9), posterior cervical rib (Fig. 18N).

*No locality data (beyond* Cynognathus *Zone Subzone A, Free State)*

BP/1/6739, partial left parietal (Fig. 11F-G).

BP/1/7340, far posterior end of right mandible (not figured).

BP/1/7234, cervical vertebra (not figured)

**B. Non-type, referred material**

Our identification of this material as *G. madiba* is much less certain than for the type material. These elements were generally found isolated and/or are not used, figured or mentioned in our morphological description. These specimens include much of material sectioned for histology.

*Farm Driefontein 11*

BP/1/6232ah, caudal vertebra.

BP/1/6232q, partial scapula.

BP/1/6232u, fragment of large left ilium.

BP/1/7129, partial humerus (Fig. 30A, 31A, B).

BP/1/7130, partial epipodial, probably radius or tibia (Fig. 30G, 32D).

BP/1/7131 partial epipodial, possibly fibula.

BP/1/7216, partial proximal tibia (Fig. 30E, 32A, B).

BP/1/7217, partial femur (Fig. 30C, 31D).

BP/1/7218, shaft region of epipodial, probably radius or tibia (Fig. 30F, 32C).

BP/1/7219, partial humerus (Fig. 30B, 31C).

BP/1/7220, girdle fragment, possibly scapula.

BP/1/7221, partial limb bone.

BP/1/7222, partial limb bone.

BP/1/7223, girdle fragment, possibly scapula.

BP/1/7224, girdle fragment.

*No locality data (beyond* Cynognathus *Zone Subzone A, Free State)*

BP/1/7134, cervical vertebra (not figured)

**LITERATURE CITED**

Brink AS (1955) Notes on some thecodonts. Navorsinge van die Nasionale Museum Bloemfontein 1:141–148.

Broili F, Schröder J (1934) Beobachtungen an Wirbeltieren der Karroo Formation. V. Über *Chasmatosaurus vanhoepeni* Haughton. Sitzungsberichte der Bayerischen Akademie der Wissenschaften, Mathematisch-naturwissenschaftliche Abteiliung 3:225–264.

Broom R (1903) On a new reptile (*Proterosuchus fergusi*) from the Karoo Beds of Tarkastad, South Africa. Annals of the South African Museum 4: 159–164.

Broom R (1905) Notice of some new reptiles from the Karoo Beds of South Africa. Records of the Albany Museum 1: 331–337.

Broom R (1906) On the remains of *Erythrosuchus africanus*. Annals of the South African Museum 5:187–196.

Broom R (1932) On some South African pseudosuchians. Annals of the Natal Museum 7:55–59.

Broom R (1946) A new primitive proterosuchid reptile. Annals of the Transvaal Museum 20:343–346.

Charig AJ, Reig OA (1970) The classification of the Proterosuchia. Biological Journal of the Linnean Society of London 2: 125–171.

Charig AJ, Sues H-D (1976) Proterosuchia. In: Kuhn O, editor*.* Handbuch der Paläoherpetologie 13. Stuttgart: Gustav Fischer. pp. 11–39.

Cheng ZW (1980) Vertebrate fossils. In: Mesozoic stratigraphy and paleontology of the Shan-Gan-Ning Basin 2. Beijing: Publishing House of Geology. pp. 114–171.

Clark JM, Welman J, Gauthier JA, Parrish JM (1993) The laterosphenoid bone of early archosauriforms. Journal of Vertebrate Paleontology 13:48–57.

Cruickshank ARI (1972) The proterosuchian thecodonts. In: Joysey KA, Kemp TS, editors. Studies in Vertebrate Evolution. Edinburgh: Oliver and Boyd. pp. 89–119.

Cruickshank ARI (1978) The pes of *Erythrosuchus africanus* Broom. Zoological Journal of the Linnean Society 62:161–177.

Gower DJ (1996) The tarsus of erythrosuchid archosaurs, and implications for early diapsid phylogeny. Zoological Journal of the Linnean Society 116: 347–375.

Gower DJ (1997) The braincase of the early archosaurian reptile *Erythrosuchus africanus*. Journal of Zoology, London 242: 557–576.

Gower DJ (2001) Possible postcranial pneumaticity in the last common ancestor of birds and crocodilians: evidence from *Erythrosuchus* and other early archosaurs. Naturwissenschaften 88:119–122.

Gower DJ (2003) Osteology of the early archosaurian reptile *Erythrosuchus africanus* Broom. Annals of the South African Museum 110: 1–84.

Gower DJ, Sennikov AG (1996) Morphology and phylogenetic informativeness of early archosaur braincases. Palaeontology 39: 883–906.

Gower DJ, Sennikov AG (1997) *Sarmatosuchus* and the early history of the Archosauria. Journal of Vertebrate Paleontology 17: 60–73.

Gower DJ, Sennikov AG (2000) Early archosaurs from Russia. In: Benton MJ, Shishkin MA, Unwin DM, Kurochkin EN, editors. The age of dinosaurs in Russia and Mongolia. Cambridge: Cambridge University Press. pp. 140–159.

Haughton SH (1924) On a new type of thecodont from the Middle Beaufort Beds. Annals of the Transvaal Museum 11:93–97.

Hoffman AC (1965) On the discovery of a new thecodont from the Middle Beaufort Beds. Navorsinge van die Nasionale Museum Bloemfontein 2:33–40.

Huene F von (1911) Über *Erythrosuchus*, vertreter der neuen reptil-ordnung Pelycosimia. Geologische und Paläontologische Abhandlungen, Neue Folge 10: 67–122.

Huene F von (1960) Ein grosser Pseudosuchier aus der Orenburger Trias. Palaeontographica Abteilung A 114: 105–111.

Hughes B (1963) The earliest archosaurian reptiles. South African Journal of Science 59:221–241.

Juul L (1994) The phylogeny of basal archosaurs. Palaeontologia Africana 31:1–38.

Kalandadze LL, Sennikov AG (1985) [New reptiles from the Middle Triassic of the southern Cis-Urals]. Paleontologicheskii Zhurnal 1985:777–784.

Klembara J, Welman J (2009) The anatomy of the palatoquadrate in the Lower Triassic *Proterosuchus fergusi* (Reptilia, Archosauromorpha) and its morphological transformation within the archosauriform clade. Acta Zoologica 90:275–284.

Nesbitt SJ (2011) The early evolution of archosaurs: relationships and the origin of major clades. Bulletin of the American Museum of Natural History 352: 1–292.

Ochev VG (1958) [New data concerning the pseudosuchians of the USSR]. Doklady AN SSSR 123: 749–751.

Ochev VG (1975) [On the proterosuchian palate]. Paleontologicheskii Zhurnal 1980: 98–105.

Ochev VG (1980) [New archosaurs from the Middle Triassic of the southern Cis-Urals]. Paleontologicheskii Zhurnal 1980: 101–107.

Ochev VG (1981) [On *Erythrosuchus* (*Garjainia*) *primus* Ochev]. Voprosy Geologii Yuzhnogo Urala I Povolzh’ya 22: 3–22.

Parrish JM (1992) Phylogeny of the Erythrosuchidae (Reptilia: Archosauriformes). Journal of Vertebrate Paleontology 12: 93–102.

Peng J-H. (1991) A new genus of Proterosuchia from the Lower Triassic of Shaanxi, China. Vertebrata PalAsiatica 29: 95–107.

Sennikov AG (1994) [The first Middle Triassic proterosuchid from eastern Europe]. Doklady RAN 336:659–661.

Sennikov AG (1995) [Early thecodonts of Eastern Europe]. Trudy Paleontologicheskogo Instituta RAN 263: 1–141.

Sennikov AG (2008) [Archosauromorpha]. In: Ivakhnenko MF, Kurochkin EN, editors. Fossil vertebrates from Russia and adjacent countries, fossil reptiles and birds part 1. Moscow: Russian Academy of Sciences Paleontological Institute. pp. 266–318.

Sereno PC (1991) Basal archosaurs: phylogenetic relationships and functional implications. Society of Vertebrate Paleontology Memoir 2:1–53.

Sereno PC, Arcucci AB (1990) The monophyly of crurotarsal archosaurs and the origin of bird and crocodile ankle joints. Neues Jahrbuch für Geologie und Paläontologie Abhandlungen 180:21–52.

Tatarinov LP (1961) Pseudosuchians of the USSR. Paleontological Journal 1961:117–132.

Welman J (1998) The taxonomy of the South African proterosuchids (Reptilia, Archosauromorpha). Journal of Vertebrate Paleontology 18:340–347.

Welman J, Flemming AF (1993) Statistical analysis of the skulls of Triassic proterosuchids (Reptilia, Archosauromorpha) from South Africa. Palaeontologia Africana 30:113–123.

Young C-C (1964) The pseudosuchians in China. Palaeontologica Sinica 151: 1–205.

Young C-C (1973) On the occurrence of *Vjushkovia* in Sinkiang. Memoirs of the Institute of Vertebrate Paleontology and Paleoanthropology, Academia Sinica 10: 38–52.
